# SCD2-mediated monounsaturated fatty acid metabolism regulates cGAS-STING-dependent type I IFN responses in CD4^+^ T cells

**DOI:** 10.1038/s42003-021-02310-y

**Published:** 2021-06-29

**Authors:** Toshio Kanno, Takahiro Nakajima, Satoru Yokoyama, Hikari K. Asou, Shigemi Sasamoto, Yasuhiro Kamii, Koji Hayashizaki, Yasuo Ouchi, Taishi Onodera, Yoshimasa Takahashi, Kazutaka Ikeda, Yoshinori Hasegawa, Yuki Kinjo, Osamu Ohara, Toshinori Nakayama, Yusuke Endo

**Affiliations:** 1 Department of Frontier Research and Development, Laboratory of Medical Omics Research, Kazusa DNA Research Institute, Kisarazu, Chiba Japan; 2 Department of Bacteriology, The Jikei University School of Medicine, Tokyo, Japan; 3 Jikei Center for Biofilm Science and Technology, The Jikei University School of Medicine, Tokyo, Japan; 4 Gene Expression Laboratory (GEL-B) Salk Institute for Biological Studies, La Jolla, CA USA; 5 Department of Regenerative Medicine, School of Medicine, Chiba University, Chuo-ku, Chiba Japan; 6 Department of Immunology, National Institute of Infectious Disease, Shinjuku-ku, Tokyo Japan; 7 Department of Applied Genomics Kazusa DNA Research Institute, Kisarazu, Chiba Japan; 8 Department of Immunology, Graduate School of Medicine, Chiba University, Chuo-ku, Chiba Japan; 9 AMED-CREST, AMED, Chuo-ku, Chiba Japan; 10 Department of Omics Medicine, Graduate School of Medicine, Chiba University, Chuo-ku, Chiba Japan

**Keywords:** Antimicrobial responses, Viral host response, T-helper 1 cells, Interferons

## Abstract

Host lipid metabolism and viral responses are intimately connected. However, the process by which the acquired immune systems adapts lipid metabolism to meet demands, and whether or not the metabolic rewiring confers a selective advantage to host immunity, remains unclear. Here we show that viral infection attenuates the expression of genes related to lipid metabolism in murine CD4^+^ T cells, which in turn increases the expression of antiviral genes. Inhibition of the fatty acid synthesis pathway substantially increases the basal expression of antiviral genes via the spontaneous production of type I interferon (IFN). Using a combination of CRISPR/Cas9-mediated genome editing technology and a global lipidomics analysis, we found that the decrease in monounsaturated fatty acid caused by genetic deletion of *Scd2* in mice was crucial for the induction of an antiviral response through activation of the cGAS-STING pathway. These findings demonstrate the important relationship between fatty acid biosynthesis and type I IFN responses that enhances the antiviral response.

## Abstract (web summary)

Kanno et al. demonstrate that decreased monounsaturated fatty acid in CD4 + T cells following *Scd2* deletion boosts the induction of the antiviral response via activation of the cGAS-STING pathway in mice. This study highlights the important interaction between fatty acid metabolism and the acquired immune response.

## Introduction

Host lipid metabolism and viral responses are intimately connected^1,2^. The changes in lipid metabolism due to viral infection can have both harmful and beneficial effects on host cells. Human cytomegalovirus alters the host cell lipid metabolism to produce long-chain fatty acids for viral replication^3^. Hansen et al. reported that the DNA virus herpes simplex type 2 induces nitro-fatty acid production, resulting in the inhibition of host cell-derived interferon (IFN) generation^4^. In contrast, it was reported that the polyunsaturated fatty acid (PUFA)-derived lipid mediator protectin D1 attenuates influenza viral replication via the RNA export machinery as a beneficial role of lipids in host defense^5^. Interestingly, in macrophages, the abrogation of the cholesterol synthesis pathway triggers the spontaneous production of type I IFN (IFN-I), resulting in the upregulation of antiviral activity^6^.

IFN-I elicits resistance to invading viral pathogens by inducing expression of a diverse range of genes related to the antiviral response, called interferon-stimulated genes (ISGs). Due to the ubiquitous expression of the IFN-I receptor (IFNAR), most cells can receive IFN-I signaling. In addition to ISGs upregulation, IFN-I has been shown to influence the ability of CD4^+^ T cells to construct follicular helper T cells and help B cells produce antibodies^7^. Although IFN-I is mainly produced by innate immune cells, such as plasmacytoid dendritic cells (pDCs) and macrophages, CD4^+^ T cells can also produce IFN-I in response to viral infection^8^.

It was recently reported that IFN-I induces changes in the core lipid metabolism that are critical for the immune function against antiviral responses^9^. IFN-I signaling increases the fatty acid oxidization (FAO) and oxidative phosphorylation (OXPHOS) required for the full activation of pDCs^9^. In macrophages, IFNβ treatment reduces the total amount of cellular cholesterol and increased the level of the antiviral lipid 25-hydroxycholesterol (25HC)^10^. 25HC restricts mammalian retrovirus infection by inhibiting viral DNA replication, viral entry and growth. Thus, the lipid metabolism and antiviral response are mutually connected in innate immune cells. However, little is known about the specific lipid metabolic regulators that control the adaptive immune cell function against virus infection.

The intracellular metabolism reportedly contributes to the regulation of proliferation, activation and memory generation of T cells^11,12^. In naïve CD8^+^ T cells, the metabolic switch from OXPHOS to glycolysis is required for effector differentiation upon antigen invasion^13^. In the context of acquired immunity, cell intrinsic lysosomal lipolysis is necessary for the generation and maintenance of memory CD8^+^ T cells^14^. CD4^+^ T cells also require lipid metabolism for their proliferation, activation and memory generation. mTOR-PPARγ axis-mediated fatty acid metabolic reprogramming is required for the early activation of CD4^+^ T cells^15^.

Acetyl-CoA carboxylase 1 (ACC1), a rate-limiting enzyme of fatty acid biosynthesis, contributes to Th17 cell differentiation in mouse and human obesity^16^. It has also been reported that each effector Th cell subset uses different chain lengths of fatty acids for their differentiation^17^. The middle- and long-chain fatty acids support Th1 and Th17 cell differentiation and short-chain fatty acids support regulatory T cell differentiation. We also previously reported that ACC1 characterizes the memory potential of individual CD4^+^ T cells^18^. ACC1^lo^ effector Th1 cells are more likely to become memory cells compared to ACC1^hi^ effector Th1 cells. Interestingly, type I IFN-related genes are upregulated in ACC1^lo^ effector Th1 cells. However, while mounting evidence indicates the importance of lipid metabolism for T cell differentiation and function, the role of lipid metabolism in the antiviral responses remains unclear in CD4^+^ T cells.

In the present study, we investigated the relationship between the fatty acid synthesis pathway and antiviral responses in CD4^+^ T cells. We found that influenza virus infection attenuated the expression of genes associated with lipid metabolism in CD4^+^ T cells. The genetic deletion or pharmacological inhibition of ACC1 resulted in a dramatic increase in ISGs expression in both mouse and human Th1 cells. Deeper analyses revealed that the ISGs expression was augmented following IFN-I production in an autocrine (and/or paracrine) fashion through IFNAR signaling. Furthermore, a mechanistic study indicated that limiting the levels of triacylglycerol containing MUFA via deletion of the stearyl-CoA desaturase (SCD) gene *Scd2* was crucial for the regulation of the T cell antiviral responses induced by the cGAS-STING signaling axis. Our data therefore indicate that changes in the MUFA metabolism in T cells during virus infection trigger cGAS-STING-mediated IFN-I-related immune responses.

## Results

### Influenza virus infection reprograms lipid metabolism in lung CD4^+^ T cells

**Fig. 1:**
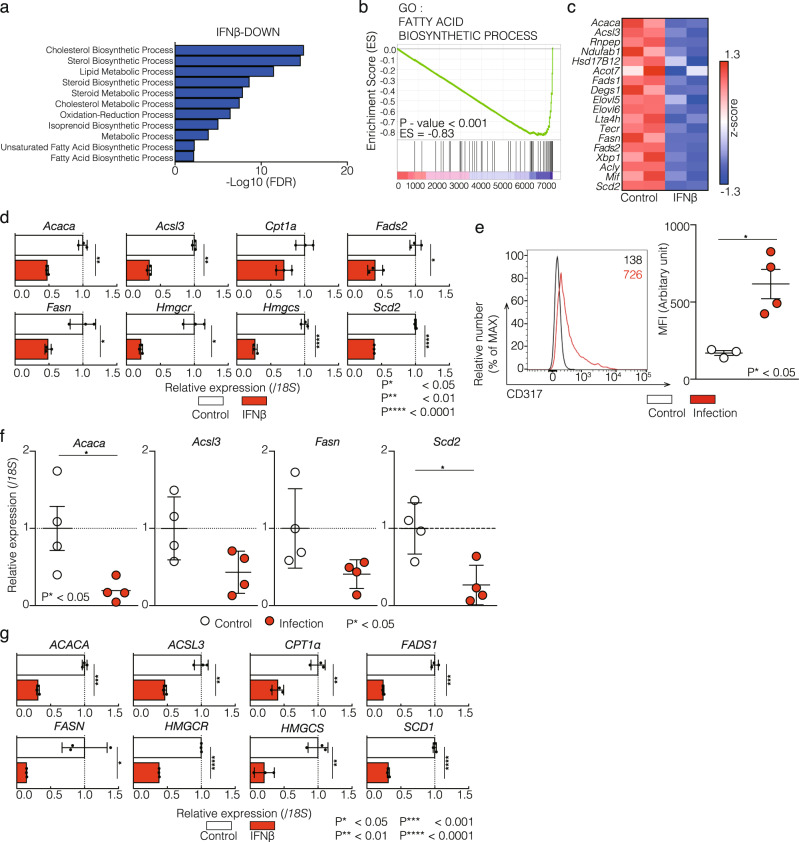
Influenza virus infection reprograms lipid metabolism in lung CD4^+^ T cells. **a** Ontology analysis of RNA-sequencing was performed in IFNβ treated Th1 cells as compared to control Th1 cells by using DAVID software (1.5-fold decrease). FDR values are on −log 10 value. (control, *n* = 2; IFNβ, *n* = 2 biologically independent sample). **b** Gene set enrichment analysis (GSEA) reveals the downregulation of the fatty acid biosynthesis genes in Th1 cells treated with IFNβ. Genes are ranked into an ordered list on the basis of fold change in control and IFNβ treated Th1 cells. (Control, *n* = 2; IFNβ, *n* = 2 biologically independent sample). **c** A heat map depicts the gene relevant to (**b**). **d** qRT-PCR analyses of the relative expression of genes relevant to lipid metabolism in mouse Th1 cells with or without IFNβ treatment. Relative expression (normalized to *18S*) with SD is shown. **e** Cell surface staining of CD317 on lung CD4^+^ T cells derived from control or X31 infected mice are shown. Mean fluorescence intensity (MFI) of CD317 are indicated (Control, *n* = 3; Infection (+), *n* = 4 biologically independent sample). **f** qRT-PCR analyses of the relative expression of fatty acid synthesis genes in lung CD4^+^ T cell derived from control or X31 infected mice. Relative expression (normalized to 18S) with SD is shown. Each dot showed averaged expression of genes in single sample (Control, *n* = 3; Infection (+), *n* = 4 biologically independent sample). **g** qRT-PCR analyses of the relative expression of genes relevant to lipid metabolism in human Th1 cells with or without IFNβ. Relative expression (normalized to *18S*) with SD is shown. Two biological replicates were performed for RNA-sequencing analysis (**a**–**c**). More than two independent experiments were performed and showed similar results (**e**, **f** = 2; **d**, **g** = 3). Three technical replicates were performed with quantitative RT-PCR and relative expression (normalized to *18S*) with SD is shown (**d**, **f**, **g**).

To elucidate the role of IFN in cellular metabolism of T cells, we first performed a RNA-sequence analysis following treatment of Th1 cells with the antiviral protein IFNβ. Gene ontology and pathway analyses using the NIAID DAVID and KEGG databases^19^ showed a significant enrichment of several functional categories, including the fatty acid biosynthetic process (Fig. 1a). A gene set enrichment analysis (GSEA) also showed the decreased expression of gene sets including the fatty acid biosynthetic process (Fig. 1b). Importantly, IFNβ treatment resulted in the decreased expression of *Acaca*, which encodes ACC1, a rate-limiting enzyme for fatty acid biosynthesis (Fig. 1c). Quantitative real-time polymerase chain reaction (qRT-PCR) also showed the decreased expression of genes related to lipid metabolism (Fig. 1d). These data suggest that IFN-I signaling suppresses the lipid metabolism in T cells during virus infection.

We next examined the expression of genes associated with lipid metabolism in lung CD4^+^ T cells following influenza virus infection (X31 strain). The expression of CD317, which is a surface protein known to restrict virus infection^8^, was increased on lung CD4^+^ T cells and comparable to that on other adaptive immune cells, such as CD8^+^ T cells and B cells (Fig. 1e, and Supplementary Fig. 1a, 1b). Splenic CD4^+^ T cells, CD8^+^ T cells and B cells showed very slight changes in the CD317 expression (Supplementary Fig. 1c). Consistent with these data, the enhanced expression of representative ISGs, such as *Ddx60*, *Ifit1*, *Mx1* and *Oas3*, was detected in lung CD4^+^ T cells obtained from infected mice (Supplementary Fig. 1d). In sharp contrast, a substantially reduced expression of genes involved in the lipid metabolism was detected in CD4^+^ T cells from X31-infected mice (Fig. 1f). Interestingly, slight changes were observed in the expression of genes related to the lipid metabolism in CD8^+^ T cells (Supplementary Fig. 1e).

To explore whether or not a similar phenomenon was observed in human CD4^+^ T cells, we treated primary human PBMC-derived CD4^+^ T cells with IFNβ. Consistent with our findings in the mouse experiment, IFNβ treatment decreased the expression of genes related to lipid metabolism (Fig. 1g).

### The fatty acid biosynthesis pathway controls the ISGs expression in CD4^+^ T cells

**Fig. 2:**
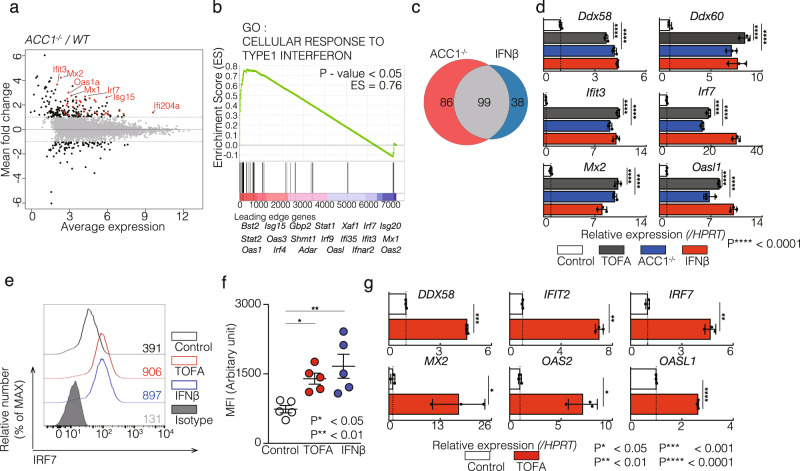
The fatty acid biosynthesis pathway controls the ISGs expression in CD4^+^ T cells. **a** A scatter plot of gene expression by RNA-sequencing (*n* = 2 per genotype) compares in wild-type and ACC1^−/−^ Th1 cells. The dashed lines indicate twofold cut-off for the difference in gene expression levels. mRNA levels showed average expression (*x*-axis) and fold change (*y*-axis) on a log2 scale. (Control, *n* = 2; ACC1^−/−^, *n* = 2 biologically independent sample). **b** GSEA analysis reveals the upregulation of the ISGs in ACC1^−/−^ Th1 cells. Genes are ranked into an ordered list on the basis of fold change in wild-type and ACC1^−/−^ Th1 cells. Genes below the picture indicate leading edge subset. **c** Venn diagram showed over 2.0-fold increased genes in ACC1^−/−^ and IFNβ-treated Th1 cells as compared to control Th1 cells. Data of IFNβ-treated Th1 cells are same as Fig. 1a. **d** qRT-PCR analyses of the relative expression of ISGs in WT and ACC1^−/−^ Th1 cells. 10 μM TOFA and 100 U/ml IFNβ were treated for 72 h. **e** Intracellular staining and flow cytometry analyzing IRF7 in Th1 cells treated with TOFA or IFNβ as in (**d**) are shown. Mean fluorescence intensity (MFI) of IRF7 is indicated. Isotype means isotype-matched control antibody. (*n* = 4 per each group biologically independent sample). **f** Comparison of IRF7 expression of five independent experiments are shown. Each dot represents one experiment. Data are means ± SD. (*n* = 5 per each group biologically independent sample). **g** qRT-PCR analyses of the relative expression of ISGs in human Th1 cells treated with or without 10 μM TOFA. Human Th1 cells were differentiated from human PBMC-derived naïve CD4^+^ T cells under Th1 skewed conditions. Relative expression (normalized to *Hprt*) with SD is shown. Two biological replicates were performed for RNA-sequencing analysis (**a**–**c**). More than three independent experiments were performed and showed similar results (**d**–**g**). Three technical replicates were performed with quantitative RT-PCR and relative expression (normalized to *Hprt*) with SD is shown (**d**, **g**).

The results in Fig. 1 prompted the hypothesis that reduced levels of fatty acid biosynthesis might be required for the induction of basal level of ISGs in CD4^+^ T cells. We used mice in which the biotin carboxyl carrier protein domain in the *Acaca* gene had been conditionally deleted in CD4^+^ T cells driven by the *Cd4* promoter (herein referred to as ACC1^−/−^)^20^. To investigate the role of ACC1 in T cell development, we analyzed thymus and spleen derived from ACC1^−/−^ mice. ACC1^−/−^ mice showed normal proportion and numbers of CD4^+^ and CD8^+^ T cells in the thymus (Supplementary Fig. 2a, upper panel), whereas the proportion and numbers of CD4^+^ and CD8^+^ T cells in the spleen was slightly reduced in ACC1^−/−^ mice compared to ACC1^+/+^ mice (Supplementary Fig. 2a, lower panel). However, we did not find significant changes in the proportion of memory phenotype CD4^+^ T cells and naïve CD4^+^ T cells between ACC1^+/+^ and ACC1^−/−^ mice (Supplementary Fig. 2b). To examine the effect of *Acaca* deletion on the expression of ISGs, we analyzed the global gene expression profiles of Th1 cells in ACC1^−/−^ mice. A total of 395 genes showed a greater than twofold change, including 185 up-regulated and 210 down-regulated genes in ACC1^−/−^ Th1 cells. Importantly, the genes concerning antiviral responses were significantly enriched in ACC1^−/−^ Th1 cells without IFN-I treatment (Fig. 2a, and Supplementary Fig. 2c). A GSEA confirmed statistically significant enrichment of IFN-I-inducible genes in ACC1^−/−^ cells (Fig. 2b). Similarly, the dramatic up-regulation of ISGs was detected when we treated Th1 cells with TOFA, an allosteric inhibitor of ACC1 (Supplementary Fig. 2d and 2e). An ontology analysis also showed that the pharmacological inhibition of ACC1 resulted in the enrichment of the pathway related to the antiviral response (Supplementary Fig. 2 f). These results indicate that the cell-autonomous basal expression of antiviral genes was induced by both genetic deletion and pharmacological inhibition of ACC1 in CD4^+^ T cells.

We next compared the gene expression profiles of ACC1^−/−^ and IFN-I treated Th1 cells, and surprisingly, 99 up-regulated genes (53.5% and 72.3% of up-regulated genes in ACC1^−/−^ and IFN-I-treated Th1 cells, respectively) were shared between the two groups (Fig. 2c). Quantitative RT-PCR analysis confirmed that the basal expression of ISGs was increased in ACC1^−/−^ or TOFA-treated cells as a comparable level to IFN-I-treated cells (Fig. 2d and Supplementary Fig. 2g). We also found the upregulation of ISGs in ACC1^−/−^ or TOFA-treated CD4^+^ T cells cultured under Th2 polarization conditions (Supplementary Fig. 2h). Similarly, the expression of ISGs was enhanced in TOFA-treated Th0, Th9, Th17 or regulatory T cells as comparable levels to those of cells treated with IFN-I. (Supplementary Fig. 2i-l). In addition, we also observed that the ISGs expression was upregulated in CD8^+^ T cells by the treatment of TOFA (Supplementary Fig. 2m). IRF7, interferon regulatory factor 7, is known as an essential transcription factor controlling the expression of ISGs for antiviral immune responses^21^. Protein expression of IRF7 was significantly upregulated by the inhibition of fatty acid biosynthesis (Figs. 2e and 2f). The basal expression level of ISGs was also increased by the inhibition of the fatty acid biosynthesis pathway in human Th1 cells (Fig. 2g). These data indicate inhibition of fatty acid synthesis increases the basal expression of ISGs in CD4^+^ and CD8^+^ T cells.

### Spontaneous production of IFNα by limiting the fatty acid biosynthesis pathway triggers a type I IFN response in CD4^+^ T cells

**Fig. 3:**
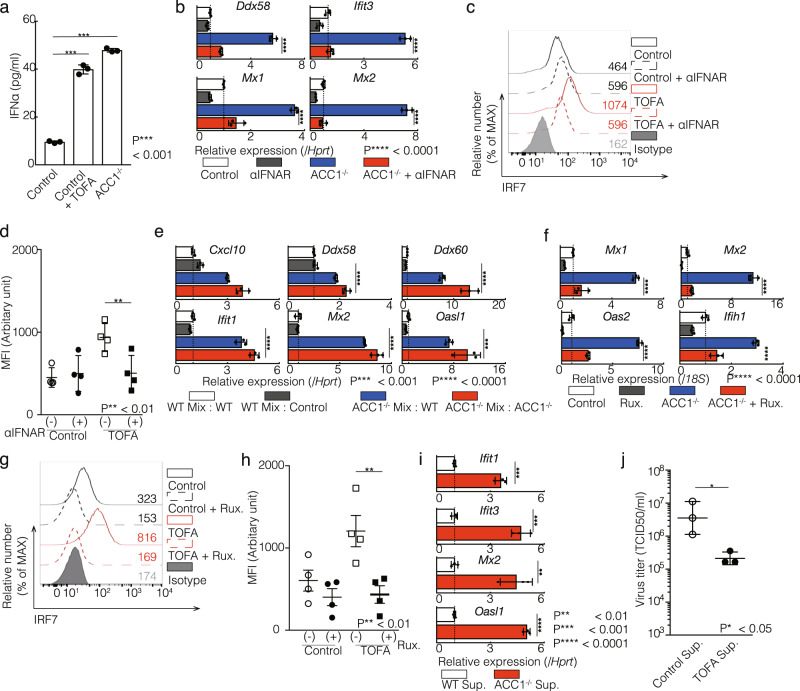
Spontaneous production of IFNα by limiting the fatty acid biosynthesis pathway triggers a type I IFN response in CD4^+^ T cells. **a** The amount of IFNα in the cell supernatant was measured by ELISA. Data are means ± SD. **b** qRT-PCR analyses of the relative expression of ISGs in WT and ACC1 KO Th1 cells. 10 μg/ml IFNAR neutralizing antibody was treated for 72 h after TCR stimulation. Relative expression (normalized to *Hprt*) with SD is shown. **c** Intracellular staining and flow cytometry analyzing of IRF7 in Th1 cells treated with IFNAR neutralizing antibody as in (**b**) are shown. Mean fluorescence intensity (MFI) of IRF7 are shown. Isotype means isotype-matched control antibody. **d** Summary data of four independent experiments of IRF7 expression are shown. Each dot represents one experiment. Data are means ± SD. (*n* = 4 per each group biologically independent sample). **e** Co-culture experiment was designed as Supplementary Fig. 3a. Co-culture was started after 48 TCR stimulation. Under the WT mix conditions, WT and littermate Th1 cells were cultured in a same well. Under the ACC1^−/−^ mix conditions, WT and ACC1^−/−^ Th1 cells were cultured in a same well. After 72 h, cells were harvested and sorted by flow cytometer based on the expression of Ly5.1(WT) and Ly5.2 (littermate or ACC1^−/−^). qRT-PCR analyses of the relative expression of ISGs in sorted Th1 cells. Relative expression (normalized to *Hprt*) with SD is shown. **f** qRT-PCR analyses of the relative expression of ISGs in WT and ACC1^−/−^ Th1 cells. 1 nM JAK inhibitor ruxolitinib was treated for 72 h. Relative expression (normalized to *18S*) with SD is shown. **g** Intracellular staining and flow cytometry analyzing of IRF7 in Th1 cells treated with JAK1 inhibitor as in (**f**) are shown. Mean fluorescence intensity (MFI) of IRF7 are shown. Isotype means isotype-matched control antibody. **h** Summary data of four independent experiments of IRF7 expression are shown here. Each dot represents one experiment. Data are means ± SD. (*n* = 4 per each group biologically independent sample). **i** MLE-15 cells were cultured with supernatant of Th1 cells for 24 h as in Supplementary Fig. 3d. qRT-PCR analysis of ISGs in MLE-15 cells was performed. Relative expression (normalized to *Hprt*) with SD is shown. Three independent experiments for each group were performed with similar results. **j** MLE-15 cells were cultured as in (**i**) and infected with ×31. Virus titers were determined 72 h p.i. (*n* = 3 per each group biologically independent sample). More than three independent experiments were performed and showed similar results (**a**–**j**). More than three technical replicates were performed with quantitative RT-PCR and relative expression (normalized to *Hprt*) with SD is shown (**a**, **b**, **e**, **f** and **i**).

To investigate the mechanism underlying the induction of ISGs by the inhibition of de novo fatty acid biosynthesis in CD4^+^ T cells, we first quantified the amount of IFNα in the Th1 cell culture supernatant without TCR re-stimulation. Significantly higher concentrations of IFNα were detected in the supernatants cultured from ACC1^−/−^ and TOFA-treated Th1 cells (Fig. 3a). Importantly, the induction of ISGs in ACC1^−/−^ Th1 cells was clearly prevented by IFNAR blockade, suggesting that the secreted IFNα might have induced the ISGs expression (Fig. 3b). We also found that the protein level of IRF7 was significantly decreased by the inhibition of IFNAR signaling in TOFA-treated Th1 cells (Fig. 3c and 3d).

We additionally investigated the influence of IFNα produced by ACC1^−/−^ Th1 cells on the induction of ISGs using co-culture systems (Supplementary Fig. 3a). Co-culture of wild-type (WT) and ACC1^−/−^ Th1 cells led to the upregulation of ISGs and IRF7 protein of co-cultured WT Th1 cells, with levels almost the same as those in ACC1^−/−^ Th1 cells (Fig. 3e, Supplementary Fig. 3b, and 3c). These results reinforced that IFNα produced by ACC1^−/−^ Th1 cells plays a role in autocrine and paracrine signaling through IFNAR to induce the expression of ISGs. Upon the binding of IFN-I on IFNAR, the JAK/STAT pathway is activated, inducing the construction of ISGF3, composed of STAT1, STAT2 and IRF9 for the production of ISGs^22^. The JAK1/2 inhibitor Ruxolitinib reduced the mRNA expression of ISGs and the protein level of IRF7 in ACC1^−/−^ or TOFA-treated Th1 cells (Fig. 3f–3h).

### Limiting fatty acid biosynthesis protects against viral infection

Our data suggested that the inhibition of the fatty acid biosynthesis pathway induced spontaneous IFNα production by CD4^+^ T cells, resulting in the ISGs upregulation in surrounding cells. These data prompted us to investigate whether or not the inhibition of fatty acid synthesis induces antiviral activity.

We collected supernatants from control, ACC1^−/−^ or TOFA-treated Th1 cells and supplemented them to MLE-15 cells, a murine lung epithelial cell line (Supplementary Fig. 3d). The expression of ISGs and IRF7 protein was strongly induced in MLE-15 cells supplemented with culture supernatant from ACC1^−/−^ or TOFA-treated Th1 cells (Fig. 3i and Supplementary Fig. 3e). Consistent with the augmentation of the ISGs expression, the supplementation of MLE-15 cells with the culture supernatant from TOFA-treated Th1 cells conferred resistance against influenza virus (Fig. 3j). A set of data indicate that spontaneous IFNα production induced by the inhibition of de novo fatty acid biosynthesis in CD4^+^ T cells can activate basal level of ISGs expression and induce antiviral responses in the surrounding cells.

### The MUFA metabolism plays a crucial role in the regulation of the type I IFN response in CD4^+^ T cells

**Fig. 4:**
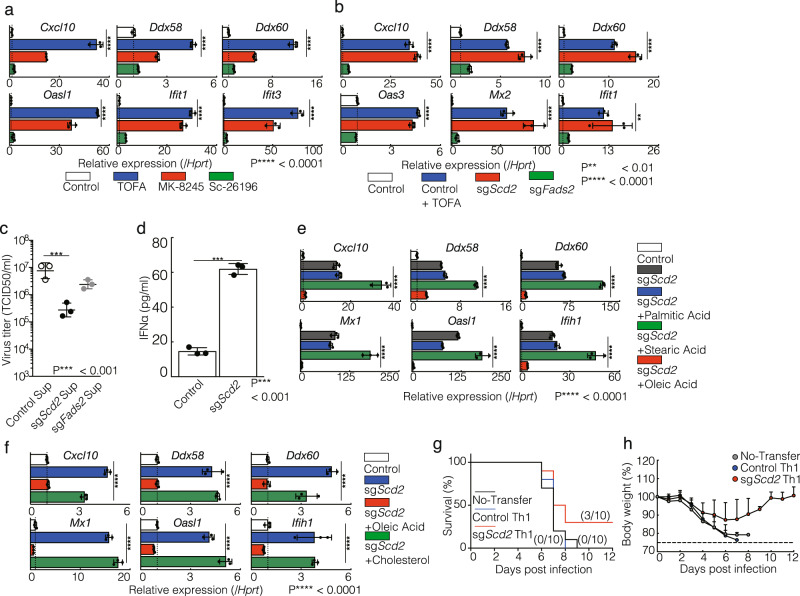
The MUFA metabolism plays a crucial role in the regulation of the type I IFN response and antiviral response in CD4^+^ T cells. **a** qRT-PCR analyses of the relative expression of ISGs in Th1 cells treated with 10 μM TOFA, 1 μM Mk-8245, or 30 μM Sc-26196. Relative expression (normalized to *Hprt*) with SD is shown. **b** qRT-PCR analyses of the relative expression of ISGs in control, sg*Scd2* and sg*Fads2* Th1 cells. Relative expression (normalized to *Hprt*) with SD is shown. **c** MLE-15 cells were cultured with culture supernatants of control, sg*Scd2* and sg*Fads2* Th1 cells and infected with x31. Virus titers were determined 72 h p.i. (*n* = 3 per each group biologically independent sample). **d** The amount of IFNα in the cell supernatant was measured by ELISA. Data are means ± SD. **e**, **f** qRT-PCR analyses of relative expression of ISGs in control, sg*Scd2* Th1 cells supplemented with 30 μM palmitic acid, 10 μM stearic acid, 30 μM oleic acid (**e**) or 10 μg/ml cholesterol (**f**) for 72 h. **g**, **h** Survival rate (**g**) or weight change (**h**) of PR8 infected mice was assessed 1 day after administrative transfer of control or sg*Scd2* Th1 cells. Relative expression (normalized to *Hprt*) with SD is shown. More than two independent experiments were performed and showed similar results (**a**–**f** = **3**). Three technical replicates were performed with quantitative RT-PCR and relative expression (normalized to *Hprt*) with SD is shown (**a**, **b**, **e**, **f**).

Since ACC1 is a rate-limiting enzyme of fatty acid biosynthesis, the inhibition of ACC1 can globally alter the cellular lipid profiles^20^. To clarify which type of fatty acids play an essential role in the regulation of ISGs induction, we focused on SCD and fatty acid desaturase (FADS), which control the production of MUFA and PUFA, respectively (Supplementary Fig. 4a). When we pharmacologically inhibited SCD enzyme, the basal expression level of ISGs was substantially induced in Th1 cells. Treatment of FADS inhibitor modestly increased basal expression level of ISGs compared to control group (Fig. 4a and Supplementary Fig. 4b).

To further evaluate the role of SCD and FADS in ISGs regulation in CD4^+^ T cells, we performed CRISPR/Cas9-mediated genome editing of *Scd2* and *Fads2*, which are preferentially expressed in Th1 cells (Supplementary Fig. 4c-f). The genetic deletion of *Scd2* (sg*Scd2*) in Th1 cells increased the basal ISGs expression almost to the same level seen in ACC1^−/−^ cells (Fig. 4b). Although the genetic deletion of *Fads2* (sg*Fads2*) resulted in the higher level of ISGs expression as compared to control Th1 cells, those expression levels were much lower than sg*Scd2* Th1 cells. Consistently, the supplementation of MLE-15 cells with the culture supernatant from sg*Scd2* Th1 cell, which contained high levels of IFNα, significantly enhanced antiviral activity against influenza as compared to control or sg*Fads2* groups (Fig. 4c and 4d). We therefore suspected that either the accumulation of saturated fatty acid (SFA) or the deficiency of MUFA caused the induction of a type I IFN response following the deletion of *Scd2*. The upregulation of ISGs expression was clearly canceled in sg*Scd2* Th1 cells by the addition of oleic acid (OA), which is a kind of MUFA (Fig. 4e). However, extrinsic SFA supplementation was not able to decrease the basal ISGs expression in sg*Scd2* Th1 cells (Fig. 4e). The supplementation of SFA into WT Th1 cell culture did not substantially increase the basal expression of ISGs (Supplementary Fig. 4g).

Reduced cholesterol biosynthesis reportedly enhances the antiviral responses in macrophages^6^. However, pharmacological inhibition of the sterol synthesis pathway showed only slight changes in the ISGs expression in Th1 cells (Supplementary Fig. 4h). In consistent with this data, the addition of cholesterol failed to suppress the basal expression of ISGs (Fig. 4f). Taken together, these findings indicate that a reduction in MUFA metabolites can induce the expression of ISGs and encourage antiviral responses in Th1 cells.

### Genetic deletion or pharmacological inhibition of SCD2 confer resistance to lethal influenza virus infection in vivo

To investigate whether the inhibition of fatty acid biosynthesis exhibits antiviral responses in vivo, mice were infected with A/Puerto Rico/8/34 (PR8), as causes more severe infection than X31 in mice. Mice infected with 5LD50 of PR8 virus were monitored for 12 days to analyze weight change and survival. Although the mice injected with control Th1 cells failed to improve the weight loss and survival rate, 3 of 10 mice in the group injected with sg*Scd2* Th1 cells survived and there was a moderate rate of weight loss on day 5 (No-Transfer: 83.3%, Control Th1 cells: 83.1%, sg*Scd2* Th1 cells: 90.5%) (Fig. 4g and 4h). Taken together, these data indicate that genetic deletion of SCD2 augmented antiviral responses and protected mice from influenza virus infection in vivo settings.

### Rewiring the MUFA metabolism triggers cGAS-STING activation in CD4^+^ T cells

**Fig. 5:**
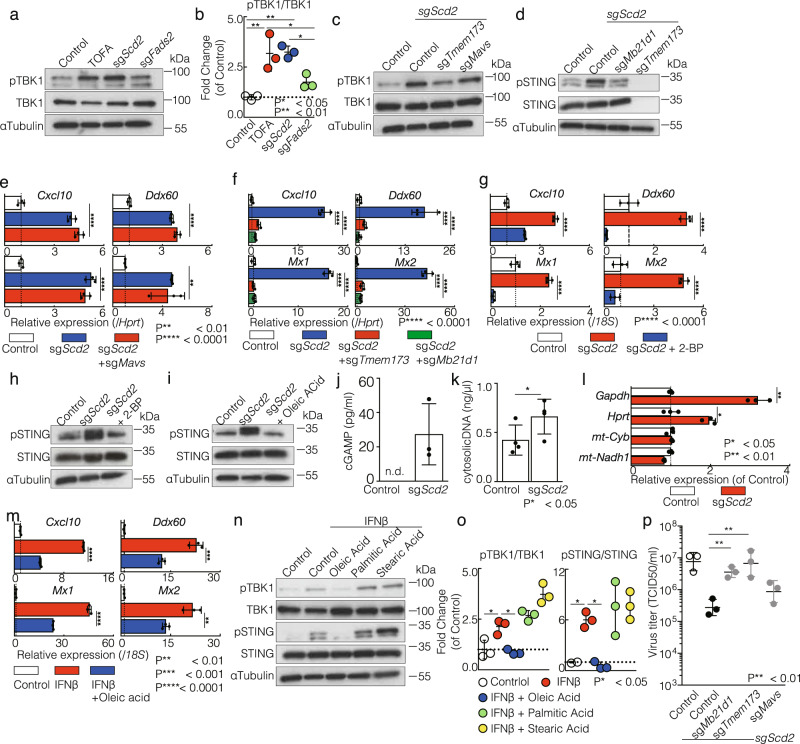
Rewiring the MUFA metabolism triggers cGAS-STING activation in CD4^+^ T cells. **a**, **b** Western blot analysis of phospho-TBK1 (pTBK1) and total TBK1 from control, TOFA-treated, sg*Scd2*, and sg*Fads2* Th1 cells (**a**). Band intensity was determined by image j and summary of three independent experiments was shown (**b**). **c** Western blot analysis of pTBK1 and total TBK1 from control, sg*Scd2*, sg*Scd2*/sg*Tmem173* DKO and sg*Scd2*/sg*Mavs* DKO of Th1 cells. **d** Western blot analysis of phospho-STING (pSTING) and total STING from control, sg*Scd2*, sg*Scd2*/sg*Mb21d1* DKO and sg*Scd2*/sg*Tmem173* DKO of Th1 cells. **e** qRT-PCR analyses of the relative expression of ISGs in control, sg*Scd2*, and sg*Scd2*/*Mavs* DKO of Th1 cells. Relative expression (normalized to *Hprt*) with SD is shown. **f** qRT-PCR analyses of the relative expression of ISGs in control, sg*Scd2*, sg*Scd2*/*sgTmem173* DKO, and sg*Scd2*/*sgMb21d1* DKO of Th1 cells. Relative expression (normalized to *Hprt*) with SD is shown. **g** qRT-PCR analyses of the relative expression of ISGs in Th1 cells. 50 μM 2-BP was treated for 72 h. Relative expression (normalized to *Hprt*) with SD is shown. **h**, **i** Western blot analysis of pSTING and total STING was performed. 50 μM 2-BP was treated for 72 h (**h**). 30 μM OA was treated for 72 h (**i**). **j** The amount of cGAMP was measured by ELISA. Data are means ± SD. **k** The amount of cytosolic DNA was measured by Qubit Fluorometer. Data are means ± SD. **l** qRT-PCR analyses of the relative expression of genomic DNA and mitochondrial DNA in cytosolic DNA derived from control Th1 or sg*Scd2* Th1 cells. SD is shown. **m** qRT-PCR analyses of the relative expression of ISGs in Th1 cells. 100 U/ml IFNβ and 30 μM oleic acid were treated for 72 h. Relative expression (normalized to *Hprt*) with SD is shown. **n**, **o** Western blot analysis of pTBK1, total TBK1, pSTING, STING from Th1 cells was performed. 100 U/ml IFNβ, 30 μM oleic acid, 30 μM palmitic acid or 10 μM stearic acid were treated for 72 h (**n**). The summary of relative intensity was shown. Band intensity was determined by image (**j**) and summary of three independent experiments was shown (**o**). **p** MLE-15 cells were cultured with culture medium of control, sg*Scd2*, sg*Scd2*/*sgMB21d1* DKO, sg*Scd2*/sg*Tmem173*, or sg*Scd2*/sg*Mavs* DKO Th1 cells and infected with x31. Virus titers were determined 72 h p.i. with similar results. More than three independent experiments were performed and showed similar results (**a**–**j** and **l**–**p** = **3**, **k** = 4). Three technical replicates were performed with quantitative RT-PCR and relative expression (normalized to *Hprt* or *18S*) with SD is shown (**e**–**g** and **l**, **m**).

Next, we sought to gain more mechanistic insight into the role of fatty acid metabolism in the induction of basal ISGs expression. To this end, we analyzed the involvement of the MAVS and STING pathways for the production of IFN-I in Th1 cells. MAVS and STING work as upstream enzymes activating TANK-binding kinase 1 (TBK1) for the production of IFN-I^23^. Higher levels of phospho-TBK1 were detected in TOFA treated and sg*Scd2* Th1 cells (Fig. 5a and Supplementary Fig. 5a). Although slight phosphorylation of TBK1 was observed in sg*Fads2* Th1 cells compared to control Th1 cells, those levels were much lower than TOFA treated and sg*Scd2* Th1 cells (Fig. 5a and 5b). We next targeted *Mavs* and *Tmem173*, which encode MAVS and STING respectively, to clarify the role of these molecules in the regulation of IFN-I production in sg*Scd2* Th1 cells. Gene targeting of STING clearly inhibited the induction of phospho-TBK1 in sg*Scd2* Th1 cells, but not *Mavs* (Fig. 5c, Supplementary Fig. 5b, and 5c). Likewise, the phosphorylation of STING was induced in sg*Scd2* Th1 cells dependent on cGAS (Fig. 5d, Supplementary Fig. 5d, and 5e). Consistent with the level of pTBK1, gene targeting of *Mavs* did not affect the basal expression levels of ISGs in sg*Scd2* Th1 cells (Fig. 5e). In contrast, double knockout of *Scd2* with *Mb21d1* or *Tmem173* failed to increase basal ISGs expression (Fig. 5f). Similar trend was observed when we treated sg*Tmem173* or sg*Mb21d1* Th1 cells with SCD inhibitor (Supplementary Fig. 5 f). It was reported that palmitoylation of STING at the Golgi is required for STING activation^24^. Thus, we next examined the effect of palmitoylation of STING on its activation using 2-bromopalmitate (2-BP), an inhibitor of STING palmitoylation and found that 2-BP suppressed STING phosphorylation and the induction of basal ISGs expression (Fig. 5g, 5h, and Supplementary Fig. 5 g). Furthermore, supplementation of OA clearly suppressed ISGs induction and STING phosphorylation (Fig. 5i and Supplementary Fig. 5 h). These results indicated that the modulation of MUFA metabolism triggers STING phosphorylation, resulting in the ISGs induction.

Next, to investigate how STING signaling was activated, we addressed whether cGAMP is involved in MUFA-mediated STING activation in CD4^+^ T cells. Although the amount of cGAMP in control Th1 cells was below the detection limit, substantial amount of cGAMP was detected in sg*Scd2* Th1 cells (Fig. 5j). Taken together, we considered the activation of STING signaling was induced by cGAS-cGAMP pathway. To support this hypothesis, we observed significantly higher amounts of cytosolic DNA in sg*Scd2* Th1 cells compared to control Th1 cells (Fig. 5k). Furthermore, the number of sequences containing *Hprt* and *Gapdh* in the genomic DNA was significantly increased in sg*Scd2* Th1 cells while the number of sequences containing *Nadh* and *cytochromeb* encoded in mitochondrial DNA was unchanged (Fig. 5l). In addition, due to the results in similar levels of mass and membrane potential of mitochondria between control and sg*Scd2* Th1 cells, we considered cGAMP was originated from genomic DNA (Supplementary Fig. 5i).

Since IFN-I treatment reduced gene expression related to lipid metabolism (Fig. 1a), we next explored whether extrinsic supplementation of fatty acids affected the expression of ISGs. Supplementation of OA moderately suppressed the ISGs upregulation induced by IFN-I treatment (Fig. 5m). Furthermore, phosphorylation of STING and TBK1 was also suppressed by supplementation of OA, but not palmitic acid or stearic acid (Fig. 5n and 5o, Supplementary Fig. 5j). We also found that treatment of IFN-I increased the amount of cytosolic DNA derived from genomic DNA (Supplementary Fig. 5k and 5l). These data indicate that the IFN-I-mediated reduction in fatty acid biosynthesis contributed at least in part, to a positive-feedback mechanism of ISGs induction in CD4^+^ T cells. Finally, we found that the antiviral responses induced by sg*Scd2* Th1 cells were significantly suppressed by gene targeting of cGAS or STING (Fig. 5p). Given these data, we concluded that rewiring the MUFA metabolism in CD4^+^ T cells triggers the activation of the cGAS-STING pathway, thereby enhancing antiviral responses.

### OA-containing TG is essential for the activation of type I IFN response in CD4^+^ T cells

**Fig. 6:**
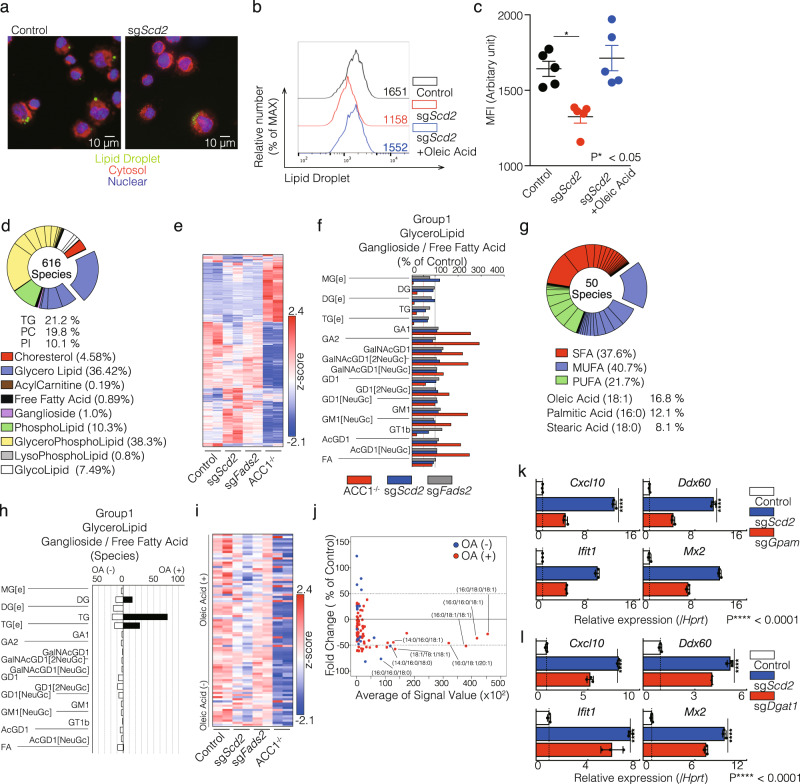
OA-containing TG is essential for the activation of type I IFN response in CD4^+^ T cells. **a** Microscopy analysis was performed using control and sg*Scd2* Th1 cells stained with lipid droplet, cytosol, and nuclear. **b** Intracellular staining and flow cytometry analyzing of lipid droplet in control and sg*Scd2* Th1 cells. Oleic acid was treated for 72 h in sg*Scd2* Th1 cells. Mean fluorescence intensity (MFI) of lipid droplet are shown. **c** Summary data of four independent experiments of lipid droplet contents are shown. Each dot represents one experiment. Data are means ± SD. **d** Pie chart showed that the ratio of lipid species control Th1 cells detected by lipidomics analysis. **e** A heat map depicts the lipid relevant to (**d**). **f** The lipidomics analysis reveals the relative contents of molecular lipid species in ACC1^−/−^, sg*Scd*2, and sg*Fads*2 Th1 cells compared to control Th1 cells. Normalized values were shown here. Two independent samples were conducted for lipidomics analysis. (*n* = 2 per each group biologically independent sample). **g** Pie chart showed that the ratio of fatty acid species incorporated into lipid detected by lipidomics analysis. **h** Species of lipid contents with or without oleic acid were shown. **i** A heat map depicts the relative contents of TGs**. j** A scatter plot depicts lipid contents of control and sg*Scd2* Th1 cells. The dashed lines indicate 50% changed line for the difference in lipid contents. The *X*-axis shows the mean value of control and sg*Scd2* Th1 cells, and the *Y*-axis shows the percentage of sg*Scd2* Th1 cells relative to control. **k** qRT-PCR analyses of the relative expression of ISGs in control, sg*Scd2*, and sg*Gpam* Th1 cells. Relative expression (normalized to *Hprt*) with SD is shown. **l** qRT-PCR analyses of the relative expression of ISGs in control, sg*Scd2*, and sg*Dgat1* Th1 cells. Relative expression (normalized to *Hprt*) with SD is shown. Two biological replicates were performed for lipidomics analysis (**d**–**j**). More than three independent experiments were performed and showed similar results (**a**, **b**, **c**, **k**, **l**). Three technical replicates were performed with quantitative RT-PCR and relative expression (normalized to *Hprt*) with SD is shown in RT-PCR (**k**, **l**).

Finally, we investigated which types of lipids are important for the regulation of basal ISGs expression when fatty acid synthesis is inhibited. Lipid droplets (LDs) are cellular organelles for the storage of neutral lipids. Although the role of LDs in adipocytes has been well studied, accumulating evidence suggests the existence of LDs in immune cells^14,25,26^. As previously described, LDs were detected in primary Th1 cells (Fig. 6a and Supplementary Fig. 6a). Intriguingly, both immunofluorescence microscopy and FACS analyses revealed that LDs of sg*Scd2* Th1 cells were smaller and lower compared to control Th1 cells (Fig. 6b, 6c, Supplementary Fig. 6a, and 6b). We therefore performed a lipidomics analysis to clarify which type of lipid was responsible for inducing basal ISGs expression in sg*Scd2* Th1 cells. A global cellular lipidomics analysis identified 616 lipid species including 177, 49, 10, 47, 216, 34, 52, 28, 3 species of glycerolipid, ganglioside, acylcarnitine, phospholipid, glycerophospholipid, lysophospholipid, glycolipid, cholesterol, and free fatty acid (Supplementary Fig. 6c). Lipid groups such as glycerophospho lipid (38.3%), glycerol lipid (36.42%) and phospholipid (10.22%) were abundant in control Th1 cells (Fig. 6d). We found that the genetic deletion of *Scd2*, *Fads2*, or *Acaca* significantly altered the overall composition of cellular lipids (Fig. 6e). Among them, the ratio of glycerophospho lipid and glycerolipid was markedly altered (Supplementary Fig. 6d). Especially, the ratio of TG, which was the most abundant lipid in control Th1 cells, was reduced by genetic deletion of *Scd2*, *Fads2*, or *Acaca* (Fig. 6f). In contrast, some lipid groups including ganglioside, PE, LPC, LPE and Sph were increased ACC1^−/−^cells (Supplementary Fig. 6e left and right). To identify the lipid species responsible for inducing ISGs, we focused on the lipid group, which of contents changed similarly in ACC1^−/−^ or sg*Scd2* Th1 cells. We found the species of decreased lipid were TG, TG [e], BMP, PC [e], and PC [sn2 + O] (Fig. 6f and Supplementary Fig. 6e). We found that OA (16.8%), palmitic acid (12.1%) and stearic acid (8.1%) were major components of cellular lipids (Fig. 6g). Due to the abundance of OA in Th1 cells and the importance of OA for basal expression of ISGs (Fig. 4e), we classified the individual lipid species based on the OA content (Fig. 6h). Notably, most TG, TG [e], and BMP contain OA, while most PC [e], and PC [sn2 + O] did not (Fig. 6h, and Supplementary Fig. 6f left). Likewise, we also observed lower occupancy of OA in lysophospholipid, and glycolipids (Supplementary Fig. 6f right). A heat map showed a marked reduction of TGs in ACC1^−/−^ Th1 cells and moderate reduction of TGs in sg*Scd2* Th1 cells compared to control Th1 cells or sg*Fads2* Th1 cells (Fig. 6i). Furthermore, deeper analysis showed that the signal value of TG containing OA was much higher than TG not containing OA, and 85% of the TGs that were reduced in sd*Scd2* Th1 cells contained OA (Fig. 6j, Supplementary fig. 6g, and 6h). Taken together, these data indicate that the amount of TG containing OA was strongly affected by the inhibition of MUFA biosynthesis *via* the deletion of *Scd2*. We also addressed whether or not a reduction in TG containing MUFA contributes to the induction of ISGs in Th1 cells. When we targeted glycerol-3-phosphate acyltransferase (GPAM), which codes the initial enzyme to catalyze the phospholipid, a modest induction of ISGs was observed (Fig. 6k). In addition, gene targeting of *Dgat1*, which codes diacylglycerol O-acyltransferase1 (DGAT1), upregulated the ISGs expression to levels comparable to those detected in sg*Scd2* Th1 cells (Fig. 6l). Although we tested the importance of lipolysis for ISGs induction, treatment of lipolysis inducing hormone isoproterenol failed to increase ISGs expression in Th1 cells (Supplementary Fig. 6i). Taken together, these results indicate that inhibition of MUFA synthesis limited the generation of TG containing OA, contributing the enhancement of basal expression of ISGs in Th1 cells.

## Discussion

In the present study, we identified a molecular mechanism to regulates antiviral genes *via* the rewiring of the MUFA metabolism in CD4^+^ T cells. We initially showed that the pharmacological inhibition or genetic deletion of ACC1 led to spontaneous IFN-I secretion, which induced the expression of ISGs in CD4^+^ T cells in both an autocrine and paracrine manner. Since the IFNα secreted by these cells can stimulate both themselves and surrounding cells, CD4^+^ T cells with a reduced activity of fatty acid biosynthesis can also exhibit an increased antiviral response to the surrounding cells. Indeed, genetic deletion or pharmacological inhibition of SCD2 increased antiviral activity both an in vivo and in vitro experimental settings. Through a combination of CRISPR/Cas9-mediated genome editing technology and a comprehensive lipidomic analysis, we found that the decreased production of MUFA incorporated into TG was crucial for the induction of an antiviral response in CD4^+^ T cells. We also found that the changes in the lipid metabolic mode led to the induction of STING and TBK1 phosphorylation *via* the activation of the cGAS-STING signal pathway. Thus, our findings provide a novel evidence that the MUFA reduction and IFN signaling pathway in CD4^+^ T cells are part of a positive-feedback mechanism.

The interrelationship between sterol biosynthesis and IFN-I signaling has been reported in macrophages^6^. The inhibition of the cholesterol biosynthesis pathway activates the IFN-I response in bone-marrow-derived macrophages (BMDMs). Furthermore, it has been also reported that the IFN response in BMDMs reduces the gene expression of *cholesterol 25-hydroxylase*, causing the suppression of *Il1* family-induced inflammation^27^. It is noteworthy that the cholesterol biosynthesis pathway is important for the induction of antiviral responses in macrophages, whereas the fatty acid biosynthesis pathway is critical for type I IFN responses in CD4^+^ T cells. Bensinger et al. mentioned that altering the balance between cholesterol biosynthesis and scavenging, rather than reducing the endogenous lipid pool size, is essential for achieving antiviral responses in macrophages^6^. In contrast, the inhibition of cholesterol biosynthesis did not affect the type I IFN response in CD4^+^ T cells. Our data further indicated that selective decrease in FA biosynthesis, especially MUFA biosynthesis, activates the type I IFN response in CD4^+^ T cells and primes these cells for antiviral immunity. Thus, although dynamic changes in cellular lipid metabolism—including fatty acid and cholesterol metabolism—are essential for antiviral immunity, the mode of acquisition and its contribution to the regulation of the type I IFN responses appear to differ among immune cell types. Further detailed studies focused on the difference in the degree of lipid metabolic dependency on the induction of IFN-I responses in acquired and innate immune cells will help expand our understanding of the crosstalk between lipid metabolism and host immune systems.

One striking point revealed in this study is that the limitation of MUFA-containing TG triggers the basal expression of ISGs in CD4^+^ T cells. When MUFA biosynthesis was inhibited by pharmacological inhibition or genetic deletion of *Scd2* in CD4^+^ T cells, cells spontaneously produced IFN-I, which strongly induced the expression of ISGs in surrounding cells. This was clearly reversed by the extrinsic supplementation of OA. While how MUFA is incorporated into TG or DG is unclear, gene targeting of *Dgat1* or *Gpam* partially augmented the expression of ISGs, implying that these enzymes further metabolize MUFA into TG to control lipid-IFN interaction in CD4^+^ T cells. Furthermore, lipidomics data showed that the levels of TG containing OA such as 16:0/18:0/18:1, 16:0/16:0/18:1, 16:0/18:1/18:1, 16:0/18:1/20:1 and 14:0/16:0/18:1 were decreased in sg*Scd2* Th1 cells. Although administration of OA in sg*Scd2* Th1 cell culture resulted in the suppressions of ISGs induction, stearic acid-treated sg*Scd2* Th1 cell modestly increased ISGs expression.

Several researchers have also reported on the relationship between lipid metabolism and antiviral responses. In pDCs, metabolic changes in FAO and OXPHOS induced by IFN-I were required for fully activation^9^. In terms of HCV infections, LDs in host cells were redistributed for the construction of a complex virus assembly. 25-hydroxychoresterol (25HC) reduces the syncytia formation of HIV, which resulted in an impairment of HIV infection. Furthermore, 25HC inhibited HIV in a humanized mouse model, and *Ch25h*^−/−^ mice was more susceptible to MHV68 lytic infection than WT mice^28^. HIV infects with CD4^+^ T cells for a long latency period. During HIV invasion, IFN-I regulates HIV infection in the initial phase^8^. In addition, HIV-1 acquires its envelop from the host cell at sites enriched in lipid raft^29^. Thus, IFN-I response through lipid metabolism in CD4^+^ T cells may be important against HIV infection. Further detailed studies focused on the role of lipid metabolism and IFN-I response in T cell during the other kinds of virus infection may lead to the discovery of novel therapeutic targets for the treatment of various viral infection.

In addition to the role of type I IFN in direct regulation of the ISGs expression and antiviral activity, it has been also reported that type I IFN limited cytotoxic activity of NK cells against antiviral CD8^+^ T cells during LCMV infection^30,31^. Thus, IFN-mediated protection of CD8^+^ T cells from NK cell cytotoxicity resulted in the proper generation of antiviral CD8^+^ T cells. Since both of CD4^+^ and CD8^+^ T cells dramatically induced ISGs expression *via* the modulation of fatty acid metabolism, this phenomenon could confer protection from NK cell-mediated cytotoxicity.

We found that cGAS-STING-mediated TBK1 activation, but not MAVS activation, is involved in the regulation of antiviral genes caused by a limitation of MUFA in CD4^+^ T cells. We considered the possibility that a reduction in MUFA might increase the availability of palmitic acid and subsequently augment the palmitoylation of STING^24^. However, supplementation of palmitic acid did not affect the inductions of ISGs in CD4^+^ T cells. We therefore believe it unlikely that changes in MUFA metabolism are significantly altering the palmitic acid availability in CD4^+^ T cells. cGAMP generated from cytosolic DNA is known to activate the cGAS-STING pathway, which triggers IFN-I production. The accumulation of cytosolic DNA reportedly enhances the IFN response in mice and humans^32,33^. Furthermore, we observed higher amount of cGAMP and cytosolic DNA originated from genome in sg*Scd2* Th1 cells, indicating that generation of cGAMP was critical for STING activation and the induction of ISGs. A recent study has demonstrated that TG synthesis is coupled with the nuclear membrane remodeling^34^. Thus, our results together with the previous study suggest that organelle interaction between LDs and nuclear membrane control cGAMP-mediated STING activation in CD4^+^ T cells.

In conclusion, our results demonstrate a novel antiviral mechanism involving the fatty acid biosynthesis pathway in CD4^+^ T cells. The decreased production of MUFA caused the activation of the cGAS-STING pathway, resulting in spontaneous IFNα production from CD4^+^ T cells. In particular, TG containing OA regulated the activation of the cGAS-STING pathway. Since inhibition of MUFA increased basal ISGs expression in tested CD4^+^ T cell subsets including Th0, Th1, Th2, Th17, Treg, and Th9 cells, these data suggested that SCD2-mediated IFN production occurs in all CD4^+^ T cell subsets. Thus, our findings shed light on novel mechanistic insights into the process of lipid metabolism that is essential for cGAS-STING activation, IFN-I response and antiviral activity in CD4^+^ T cells. Our results underscore the need to understand the role of lipids in the enhancement of basal ISGs expression and the lipid environment that favors host cell defenses.

## Methods

### Mice

The animals used in this study were backcrossed to C57BL/6 mice ten times. *Acaca*^fl/fl^ mice were crossed with CD4-cre mice (Jackson Laboratory) and were maintained on a C57BL/6 background. BALB/c and C57BL/6 mice were purchased from CLEA Japan. Ly5.1 mice were from Sankyo Laboratories. All mice were used at 6–8 weeks old and were maintained under specific-pathogen-free conditions. Animal procedures were approved by Animal Ethics Committee of the National Institute of Infectious Diseases, Japan. Animal care was conducted in accordance with the guidelines of Chiba University, KAZUSA DNA research institute, and the Institutional Animal Care and Use Committee of the National Institute of Infectious Diseases, Japan.

### Cell preparation

Splenic naïve CD4^+^ T cells were obtained by the negative selection using the Mojo Sort Mouse CD4 T Cell Isolation Kit (Biolegend) and positive selection using CD62L MicroBeads, mouse (Miltenyi Biotec). Splenic naïve CD8^+^ T cells were collected using the Naïve CD8^+^ T cells isolation kit (Miltenyi Biotec). Naïve CD4^+^ T cells and naïve CD8^+^ T cells were plated onto 24-well tissue culture plates (Costar) pre-coated with 1 μg/ml anti-CD3 antibody (clone 145-2C11, Biolegend) with 1 μg/ml anti-CD28 antibody (clone 37.51, BioLegend). Th1 cell cultures contained IL-2 (15 ng/ml), recombinant mouse IL-12 (10 ng/ml) (WAKO) and anti-IL-4 antibody (1 μg/ml) (BD Biosciences). Th2 cell cultures contained IL-2 (15 ng/ml), recombinant mouse IL-4 (10 ng/ml) (WAKO) and anti-IFNγ antibody (1 μg/ml) (BD Biosciences). Th0 cell cultures contained IL-2, anti-IL-4 antibody and anti-IFNγ antibody. Th0 cell cultures contained IL-2, anti-IL-4 antibody and anti-IFNγ antibody. Th9 cell cultures contained IL-2, recombinant mouse IL-4, TGFβ (10 ng/ml) and anti-IFNγ antibody. Th17 cell cultures contained IL-6 (10 ng/ml)(BD biosciences), TGFβ (1 ng/ml), anti-IL-2 antibody, anti-IL-4 antibody and anti-IFNγ antibody. Regulatory T cell cultures contained IL-2, TGFβ (10 ng/ml), anti-IL-2 antibody, anti-IL-4 antibody and anti-IFNγ antibody. Naïve CD8^+^ T cells cultures contained IL-2. Recombinant mouse IFNβ (Biolegend) were dissolved in PBS supplemented with 3% BSA. αIFNAR (clone MAR1-5A3, Leinco) were treated with 1 μg/ml. TOFA, MK-8245, Sc-26196, and Ruxolitinib were dissolved in DMSO to a final concentration of 10 μM, 1 μM, 30 μM, 100 nM respectively.

### Co-culture experiments

Naïve CD4^+^ T cell derived from Ly5.1 mouse, Ly5.2 mouse, and Ly5.2 CD4 Cre ACC1^−/−^ mouse were cultured in the presence of plate-bound anti-CD3 antibody, anti-CD28 antibody, IL-2 (15 ng/ml), and recombinant mouse IL-12 for 48 h. Activated Ly5.2 ACC1 CD4^+^ T cell or Ly5.2 ACC1 KO Th1 cells were then co cultured with Ly5.1 CD4^+^ T cells for 72 h.

### Medium transfer experiments

Naïve CD4^+^ T cell were cultured described as **Cell Preparation** with or without TOFA. On day 5, supernatant of culture medium was harvested. After 48 h incubation of MLE-15 cells with culture supernatant, MLE-15 cells were harvested for FACS analysis and RNA isolation.

### Infection

Influenza virus was diluted to the appropriate concentration in PBS supplemented with 3% BSA. MLE-15 cells prepared described as **Medium transfer experiments** were infected with influenza virus for 3 day at 34 °C. Then, MLE-15 cells were fixed with 4% PFA and stained with amido black solution. TCID50 was calculated as described previously^35^.

### Cas9 mediated-genome-editing

The short guide RNA was designed using the online tool provided by CHOPCHOP (http://chopchop.cbu.uib.no)^36^. Freshly isolated naïve CD4 T cells were activated with plate bound anti-CD3 and CD28 antibodies. Cas9 proteins were prepared immediately before experiments by incubating 1 μg Cas9 with 0.3 μg sgRNA in transfection buffer at room temperature for 10 min. 24 h after T cell activation, these cells were electroporated with a Neon transfection kit and device (Thermo).

### In vivo experiment

The influenza A virus X31 (H3N2) were grown in a 10-d-old embryonated hen eggs and were purified through a 10–50% sucrose gradient as described^35^. BALB/c were anesthetized by i.p. injection with sodium pentobarbital and inoculated intra-nasally with 5 lethal dose50 (LD50) of influenza virus X31 in a volume of 20 μL. mRNA expression of lipid enzymes or cell surface profiles of CD317 on T cells were analyzed on day 6 after X31 infection. To investigate antiviral activity, influenza A virus A/Puerto Rico/8/34 (H1N1) was used. BALB/c were anesthetized by i.p. injection with midazolam, domitor, and butorphanol tartrate. In the administrative transfer experiment, 5 × 10^6^ of control Th1 or sg*Scd2* Th1 cells suspended in 30 μl were administered intranasally the day before infection. Infected mice were weighed daily, with 75% weight loss as the criterion for euthanasia. After three kinds of mixed anesthesia, antisedan was also administered intraperitoneally in all experiments.

### Isolation of lymphocytes from lung tissues

Mice were euthanized by anesthesia and their lungs were perfused with 5 mL cold PBS solution through the right ventricle. Lungs were transferred to a tube containing ice-cold digestion buffer [RPMI 1640 supplemented with collagenase type III (200 U/mL; Worthington) and DNase I (200 μg/mL; Sigma-Aldrich)]. Individual lungs were dissociated in 3 mL digestion buffer using a GentleMACS tissue dissociator (Miltenyi Biotec). This was followed by incubation for 30 min at 37 °C with frequent agitation and a final GentleMACS dissociation. Lung mononuclear cells were separated by centrifugation on Percoll (GE Healthcare).

### ELISA for the measurement of cytokine or cGAMP concentration

Cells were cultured as described in section of Cell Preparation. After collecting the samples, centrifugation was performed. The supernatant was used as the sample for IFNα and the cells were used as the sample for cGAMP. The concentration of IFNα or cGAMP was assessed by Mouse IFN Alpha All Subtype ELISA Kit (PBL Assay Science) or by 2′3′ – cGAMP ELISA kit (Cayman) as according to the manufacture’s protocol.

### Quantitative real-time PCR

Total RNA was isolated with the TRIzol reagent (Invitrogen). cDNA was synthesized with an oligo (dT) primer and Superscript II RT (Invitrogen). Quantitative RT-PCR was performed with the Applied Biosystems StepOnePlus^TM^ Real-Time PCR Systems as described previously. Primers and Roche Universal probes were purchased from Sigma and Roche, respectively. Gene expression was normalized with the *Hprt* mRNA signal or the *18S* ribosomal RNA signal.

### Viral genome detection

A lobe of the lung was frozen in liquid nitrogen and then crushed to make an RNA sample. Purified RNA was prepared as described in section of Quantitative Real-Time PCR. One-step RT-PCR was conducted using the One-Step TB Green PrimeScript RT-PCR Kit (Takara) according to the manufacture’s protocol. The sequence of primer is as follows.

*NP* FW: 5′-CAGCCTAATCAGACCAAATG-3′

*NP* RV: 5′-TACCTGCTTCTCAGTTCAAG-3′

### RNA-sequencing and bioinformatic analysis

Total cellular RNA was extracted with TRIzol reagent (Invitrogen). For cDNA library construction, we used the TruSeq RNA Sample Prep Kit v2 (Illumina) according to the manufacturer’s protocol. Sequencing the library fragments was performed on the HiSeq 2500 system. For data analysis, read sequences (50 bp) were aligned to the mm10 mouse reference genome (UCSC, December 2011) using Bowtie (version 0.12.8) and TopHat (version 1.3.2). Fragments per kilobase of exon per million mapped reads (FPKM) for each gene were calculated using Cufflinks (version 2.0.2). Genes with an absolute FPKM of >1 (mean from duplicate samples) were defined as expressed genes. Gene set enrichment analysis (GSEA) was performed to determine the statistical significance of the enrichment of known transcriptional signatures in a ranked list of genes^37,38^. We used 2178 gene sets from the Molecular Signature Database C5 version 3.0.

Differentially expressed genes (fold changes ≥ 2 in ACC1 KO and TOFA -treated Th1 cells or fold changes ≤ 1.5 in IFNβ-treated Th1 cells) were performed to analyze for GO biological processes using DAVID.

### Lipidomics analysis

Unbiased metabolic profiling was performed by KAZUSA DNA Research Institute using control, ACC1^−/−^, sg*Scd2*, or sg*Fads2* Th1 cells. At the end of culturing, 10 million cells were spun down and the pellets were washed with PBS (−). After treatment of mixture of chilled methanol (1:13, v/w) and methyl tert-butyl ether, samples were mixture by vigorous vortex and sonication. The required water of water (1:2.5, v/w) was added into sample before vigorous vortex. After the centrifugation, upper layer was collected for LC/MS.

### FACS analysis

Preparation of single cell suspension of mouse lung was performed as described previously^39^. Saline-perfused lung were finely minced and digested for 1 h at 37 °C in a solution of 400 U/ml collagenase III and 0.2 mg/ml DNase. Then, leukocytes were enriched by Percoll (GE Healthcare) density centrifugation. FACS staining was performed with anti-TCRβ PE, anti-CD4 FITC, anti-CD8 APC, and anti-CD45 brilliant violet 786 for single cell sorting. For the comparison of CD317 expression, anti-TCRβ PE, anti-CD4 brilliant violet 650, anti-CD19 FITC, anti-CD45 brilliant violet 786, anti-CD317 brilliant violet 421, and anti-B220 PE-Cy7 was used for cell staining.

For IRF7 staining, sample preparation was conducted with Lyse/Fix buffer (BD) and Perm buffer III (BD) according to the manufacture’s protocol. Cells were stained with anti-IRF7 PE for 45 min in the dark. MLE-15 cells were stained with anti-CD317 Alexa fluor 647 for 30 min in the dark. Flow cytometric data were analyzed with Flowjo software.

### Human T-cell cultures

Whole blood was obtained from healthy donor volunteers with consent given. Human CD4^+^ T cells were collected by a Ficoll gradient. Using Naive CD4 + T Cell Isolation Kit II (Miltenyi), Human Naïve CD4^+^ T cells were purified. Human Naïve CD4^+^ T cells were plated onto 48-well plate (Costar) pre-coated with 1 μg/ml anti-CD3 (clone OKT3) with 1 μg/ml anti-CD28 (clone CD28.2) antibody. Th1 cell cultures contained IL-2 (15 ng/ml), IL-12 (15 ng/ml), and anti-IL-4 antibody (1 μg/ml). All patients signed informed consent forms, and the study was approved by the ethics committee of the KAZUSA DNA Research Institute (authorization #2020-01).

### Immunofluorescence microscopy

The cells were fixed with 4.0% paraformaldehyde for 10 min, before permeabilized with 0.1% Triton X-100 in PBS for 10 min on ice. After then, sample was blocked with 3% BSA in PBS for 15 min and stained with indicated reagents for 30 min in the dark.

### Immunoblotting

Immunoblotting was performed as described previously^39^. Briefly, cytoplasmic extracts and nuclear extracts were prepared using NE-PER Nuclear and Cytoplasmic Extraction Reagent (Thermo Fisher Scientific). The antibodies used for the immunoblot analysis were anti-SCD2 (Santa cruz), anti-cGAS (Cell signaling), anti-MAVS (Cell signaling), anti–STING (Cell signaling), anti–phosphoSTING (Ser365) (Cell signaling), anti–TBK1 (Cell signaling), anti–phosphoTBK1 (S172) (Cell signaling) and anti-Tublin (Thermo).

### Isolation of cytosolic DNA

Cytosol fraction was prepared with Mitochondria/Cytosol Fractionation Kit (Abcam) according to the manufactured protocol. Briefly, after the treatment of RNaseA, cytosolic DNA was purified from cytosolic fraction with QIAquick Gel Extraction Kit (QIAGEN) and quantified using Qubit dsDNA HS Assay kit (Invitrogen) according to the manufactured protocol. Real-time PCR was conducted with TB Green Premix Ex Taq II (Takara).

### Primer sequences for real-time PCR

Mouse (Primer for cDNA)

*18S* FW: 5′-AAATCAGTTATGGTTCCTTTGGTC-3′

*18S* RV: 5′-GCTCTAGAATTACCACAGTTATCCAA-3′

*Acaca* FW: 5′-CCAATGGTTGAGTGGGTTTT-3′

*Acaca* RV: 5′-CGGGTATTCCCCCTAACCT-3′

*Acsl3* FW: 5′-CGGCTGTCTGAAGATCATTG-3′

*Acsl3* RV: 5′-TTCAAAGCTGCCTCTACTTTCC-3′

*Cpt1a* FW: 5′-GACTCCGCTCGCTCATTC-3′

*Cpt1a* RV: 5′-TCTGCCATCTTGAGTGGTGA-3′

*Cxcl10* FW: GCTGCCGTCATTTTCTGC

*Cxcl10* RV: TCTCACTGGCCCGTCATC

*Ddx58* FW: GAAGATTCTGGACCCCACCT

*Ddx58* RV: TGAATGTACTGCACCTCCTCAT

*Ddx60* FW: TGAAAGCCTGGAAGGAACAT

*Ddx60* RV: GCTATGCTTAAGTCTTTCGTGGTT

*Fads2* FW: 5′-CGGGAGAAGATGCTACGG-3′

*Fads2* RV: 5′-TTCAAGAACTTGCCCACGA-3′

*Fasn* FW: 5′-GCTGCTGTTGGAAGTCAGC-3′

*Fasn* RV: 5′-AGTGTTCGTTCCTCGGAGTG-3′

*Hmgcr* FW: 5′-CGTAAGCGCAGTTCCTTCC-3′

*Hmgcr* RV: 5′-TTGTAGCCTCACAGTCCTTGG-3′

*Hmgcs* FW: 5′-GGTCTGATCCCCTTTGGTG-3′

*Hmgcs* RV: 5′-TGTGAAGGACAGAGAACTGTGG-3′

*Hprt* FW: 5′-TCCTCCTCAGACCGCTTTT-3′

*Hprt* RV: 5′-CCTGGTTCATCATCGCTAATC-3′

*Ifih1* FW: 5′-CTTGTCACGAACGAGATAGCC-3′

*Ifih1* RV: 5′-CCAGGACATACGTGCTTTCA-3′

*Ifit3* FW: 5′-TGAACTGCTCAGCCCACA-3′

*Ifit3* RV: 5′-AGAGATTCCCGGTTGACCTC-3′

*Mx1* FW: 5′-TTCAAGGATCACTCATACTTCAGC-3′

*Mx1* RV: 5′-GGGAGGTGAGCTCCTCAGT-3′

*Mx2* FW: 5′-CAGTTCCTCTCAGTCCCAAGAT-3′

*Mx2* RV: 5′-TGCGGTTGTGAGCCTCTT-3′

*Oas2* FW: 5′-TAGACCAGGCCGTGGATG-3′

*Oas2* RV: 5′-GTTTCCCGGCCATAGGAG-3′

*Oasl1* FW: 5′-GGCCAACCAGTGTCTGAAA-3′

*Oasl1* RV: 5′-TGGATATCGGGTGCTCTCTT-3′

*Scd2* FW: 5′-TGGTTTCCATGGGAGCTG-3′

*Scd2* RV: 5′-TTGATGTGCCAGCGGTACT-3′

Mouse (Primer for genomic DNA or mitochondrial DNA)

*Gapdh* FW: 5′-CAGCCTAATCAGACCAAATG-3′

*Gapdh* RV: 5′-TACCTGCTTCTCAGTTCAAG -3′

*Hprt* FW: 5′-CAGCCTAATCAGACCAAATG-3′

*Hprt* RV: 5′-TACCTGCTTCTCAGTTCAAG-3′

*mt-Nadh1* FW: 5′-CAGCCTAATCAGACCAAATG-3′

*me-Nadh1* RV: 5′-TACTACCTGCTTCTCAGTTCAAG-3′

*mt-Cyb* FW: 5′-CAGCCTAATCAGACCAAATG-3′

*mt-Cyb* RV:5′-TACCTGCTTCTCAGTTCAAG-3′

Human (Primer for cDNA)

*18* *S* FW: 5′-CCGATTGGATGGTTTAGTGAG-3′

*18* *S* RV: 5′-AGTTCGACCGTCTTCTCAGC-3′

*DDX58* FW: 5′-TGTGGGCAATGTCATCAAAA-3′

*DDX58* RV: 5′-GAAGCACTTGCTACCTCTTGC-3′

*HPRT* FW: 5′-TGACCTTGATTTATTTTGCATACC-3′

*HPRT* RV: 5′-CGAGCAAGACGTTCAGTCCT-3′

*IFIT1* FW: 5′-CCTCCCTGGAAAATCTAGGC-3′

*IFIT1* RV: 5′-TCCAGACTATCCTTGACCTGATG-3′

*IRF7* FW: 5′-AGCTGTGCTGGCGAGAAG-3′

*IRF7* RV: 5′-CATGTGTGTGTGCCAGGAAT-3′

*MX2* FW: 5′-CTGGAGGCACTGTCAGGAGT-3′

*MX2* RV: 5′-CGGACACCTGGTTACGATTC-3′

*OAS2* FW: 5′-CCTGCCTTTAATGCACTGG-3′

*OAS2* RV: 5′-ATGAGCCCTGCATAAACCTC-3′

*OASl1* FW: 5′-TTGTGTCAGAAAACAGCTCAAAA-3′

*OASl1* RV: 5′-GCAACGATGTCCCATCTGTA-3′

### Primer sequences for CRISPR-Cas9 mediated gene editing

sg*cGas*: 5′- ACGCAAAGATATCTCGGAGGCGG-3′

sg*Dgat1*: 5′- CAGAGGCGGAACATCCGCAGGGG-3′

sg*Fads2*: 5′- ACAACCTGCGCACCGACCGGTGG-3′

sg*Gpam*: 5′- TAAAGGCCATACGCACCCCATGG-3′

sg*Mavs*: 5′- TTTGGTTGCTGGACCCTCCGGGG-3′

sg*Scd2*: 5′- AACCAGTGTGATCCCGTACAAGG-3′

sg*Tmem173*: 5′- TGAGGGCTACATATTTGGAGCGG-3′

### Statistics and reproducibility

Data are expressed as mean ± SD or mean ± SEM. The data were analyzed with the Graphpad Prism software program (version 7). Differences were assessed using unpaired two-tailed student *t* tests or one-way anova followed by tukey’s multiple comparisons test. Differences with *P* values of <0.05 were considered to be significant. Sample size for animal studies was chosen based on prior experience with similar models of X31 infection. No data were excluded from the analysis of experiments. Mice were commercially sourced and randomized into experimental groups upon arrival, and all animals within a single experiment were processed at the same time. For cell sorting and RNA-sequencing analysis, the investigator was blinded. Data display similar variance between groups and are normally distributed where parametric tests are used.

### Reporting summary

Further information on research design is available in the Nature Research Reporting Summary linked to this article.

## Supplementary information

Peer Review File

Supplementary Information

Description of Additional Supplementary Files

Supplementary Data 1

Supplementary Data 2

Reporting Summary

## Data Availability

The RNA seq data have been deposited in the Gene Expression Omnibus at NCBI (https://0-www-ncbi-nlm-nih-gov.brum.beds.ac.uk/geo/) under accession number GSE173728. Uncropped western blot images are presented in Supplementary Fig. 7. All source data underlying the graphs and charts shown in the main and supplementary figures are presented in Supplementary Data 1. Lipidomics source data are presented in Supplementary Data 2.
