# Supplementary material for: SCD2-mediated monounsaturated fatty acid metabolism regulates cGAS-STING-dependent type I IFN responses in CD4+ T cells
Source: Commun Biol. 2021 Jun 29;4:820. doi: 10.1038/s42003-021-02310-y (PMC8242023; doi:10.1038/s42003-021-02310-y)
Supplement: Supplementary file 2 — Supplementary Information [file 42003_2021_2310_MOESM2_ESM.pdf]

Supplemental Information

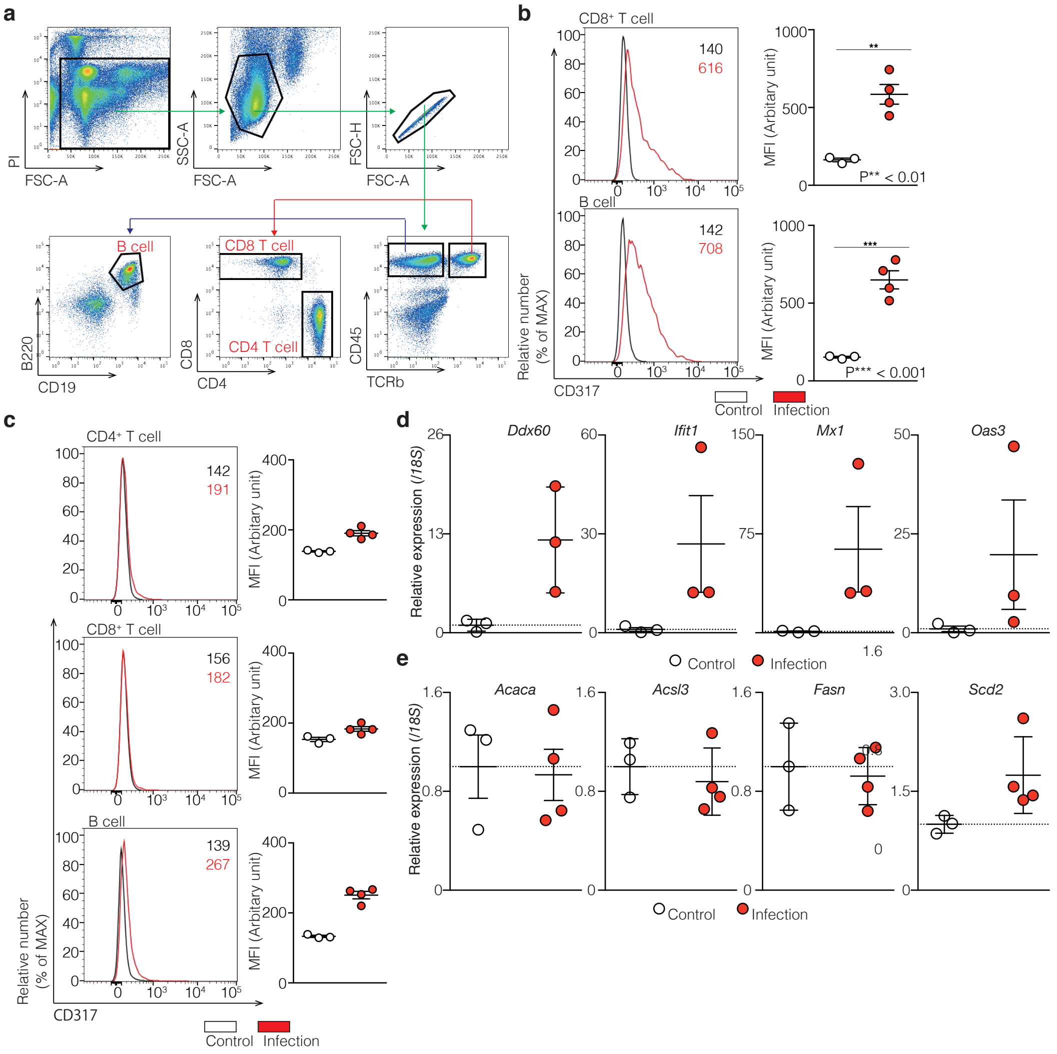

Supplementary Fig.1 Type I IFN changes the expression of genes related to lipid synthesis in mouse and human CD4⁺ T cell related to Fig.1.

a, Gating strategy of FACS analysis are shown here. **b**, **c**, Cell surface staining and flow cytometry analyzing of CD317 on lung CD8⁺ T cells and B cells (**b**) or splenic CD4⁺ T cells, CD8⁺ T cells and B cells (**c**) derived from control or X31 infected mice were shown. Mean fluorescence intensity (MFI) of CD317 are indicated. (Control, n=3;

Infection (+), n=4 biologically independent sample). **d**, qRT-PCR analyses of the relative expression of ISGs in lung CD4$^+$ T cell derived from control or X31 infected mice. Relative expression (normalized to *18S*) with SD is shown. Each dot shows averaged expression of genes in single sample. (n=3 per each group biologically independent sample).

e, qRT-PCR analyses of the relative expression of ISGs in lung CD8$^+$ T cell derived from control or X31 infected mice. Relative expression (normalized to *18S*) with SD is shown. Each dot shows averaged expression of genes in single sample. (Control, n=3; Infection (+), n=4 biologically independent sample). Two independent experiments were performed and showed similar results (**d**, **e**).

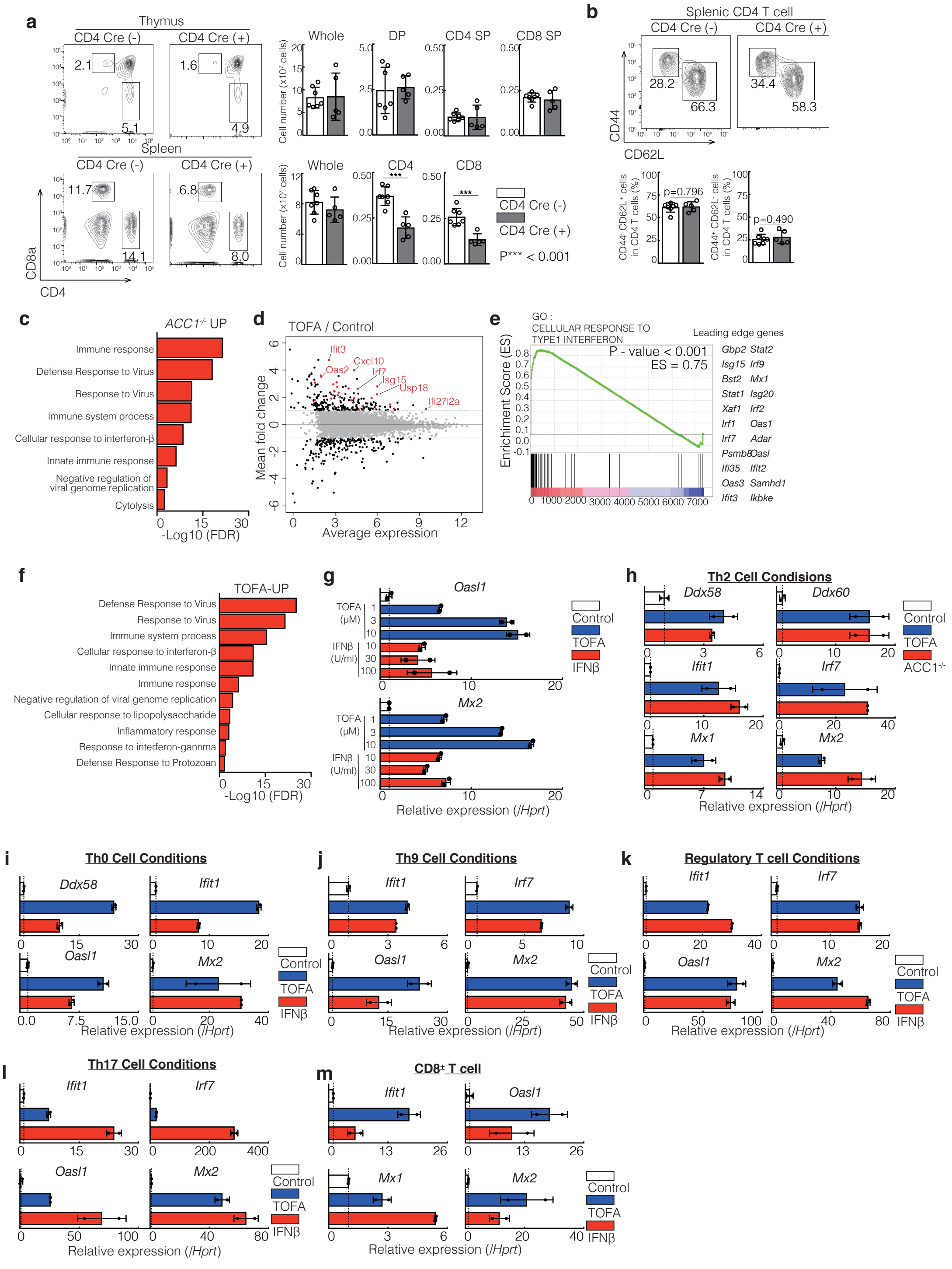

Supplementary Fig.2 Pharmacological and genetic inhibition of fatty acid synthesis pathway induces gene expression of ISGs related to Fig.2.

a, Cell surface staining profiles (CD4/CD8) of CD4-Cre (-) *Acaca*$^{fl/fl}$ or CD4-Cre (+) *Acaca*$^{fl/fl}$ thymocytes (Upper) or splenocytes (Lower). The summary data for the cell surface staining profiles (CD4 and CD8) in thymocytes and splenocytes was shown. **b** Cell surface staining profiles (CD62L/CD44) on splenic CD4^{+} T cells from CD4-Cre (-) *Acaca*$^{fl/fl}$ or CD4-Cre (+) *Acaca*$^{fl/fl}$ mice. The graph is a summary of two independent experiments. **c**, Ontology analysis of RNA-sequencing was performed in ACC1$^{-/-}$ Th1 cells as compared to littermate Th1 cells by using DAVID software (2.0-fold increase). The data used is the same as Fig. 2**a**. FDR value are on - log 10 value (n=2 per each group biologically independent sample). **d**, A scattered plot of genes expression by RNA-sequencing (n=2) compares in Th1 cells with or without TOFA treatment. The dashed lines indicate two-fold cut-off for the difference in gene expression levels. mRNA levels show average expression (x axis) and fold change (y axis) on a log2 scale. (n=2 per each group biologically independent sample). **e**, GSEA analysis revealed the upregulations of ISGs in TOFA-treated Th1 cells. Genes are ranked into an ordered list on the basis of fold changes in Th1 cells treated with or without TOFA. Gene below the picture indicates leading edge subset. Data represents the average of two independent experiments. (n=2 per each group

biologically independent sample). **f**, Ontology analysis of mRNA-seq using DAVID software was performed for over 2.0-fold increased genes in TOFA treated Th1 cells compared to control Th1 cells. The data used was same as supplementary Fig. 2**d**. FDR values are on - log 10 value. (n=2 per each group biologically independent sample). **g**, qRT-PCR analyses of the relative expression of ISGs in Th1 cells treated with indicated concentration of TOFA or IFNβ for 72 hours. Relative expression (normalized to *Hprt*) with SD is shown. **h**, qRT-PCR analyses of the relative expression of ISGs in WT and $ACC1^{-/-}$ Th2 cells. 10 μM TOFA were treated for 72 hours. Relative expression (normalized to *Hprt*) with SD is shown. **i - l**, qRT-PCR analyses showed the relative expression of ISGs in $CD4^{+}$ T cells under Th0 (**i**), Th9 (**j**), regulatory T cell (**k**), Th17 (**l**) conditions. TOFA or IFNB was treated for 72 hours. Relative expression (normalized to *Hprt*) with SD is shown. **m**, Expression levels of ISGs were quantified by RT-PCR. $CD8^{+}$ T cells were collected from the spleen, and drugs were treated for 72 hours. Three independent experiments for each group were performed with similar results. (n=3 per each group technically independent sample). Two biological replicates were performed for RNA-sequencing analysis (**c**-**f**). More than two independent experiments were performed and showed similar results (**a, b = 2**, **g-m = 3**). Three technical replicates were performed with quantitative RT-PCR (**g-m**).

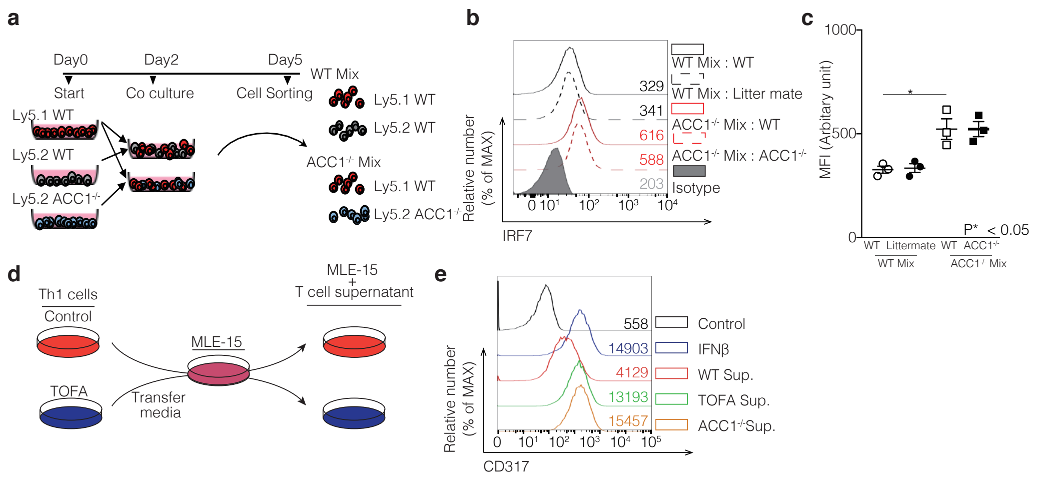

Supplementary Fig.3 IFNα produced by Th1 cells with lower activity of fatty acid synthesis acts in a paracrine manner related to Fig.3.

a, Scheme of co-cultured experimental systems are shown. **b**, Intracellular staining and flow cytometry analyzing IRF7 in Th1 cells cultured as in supplementary Fig. 3**a.** Mean fluorescence intensity (MFI) of IRF7 is indicated. Isotype means isotype-matched control antibody**. c**, Comparison of IRF7 expression of three independent experiments is shown. Each dot represents one experiment. Data are means ± SD. (n=3 per each group biologically independent sample)**. d**, Scheme of experimental systems of MLE-15 stimulated with Th1 cell culture supernatant. **e**, Cell surface staining of CD317 on MLE-15 cells cultured as in **d** are shown. 100 U/ml IFNβ were treated with MLE-15 cells for 48 hours. Two independent experiments for each group were performed with similar results. (n=2 per each group biologically independent sample). Three independent experiments were performed with co-

cultured experiment (**b**). Two independent experiments were performed and showed similar results (**e**).

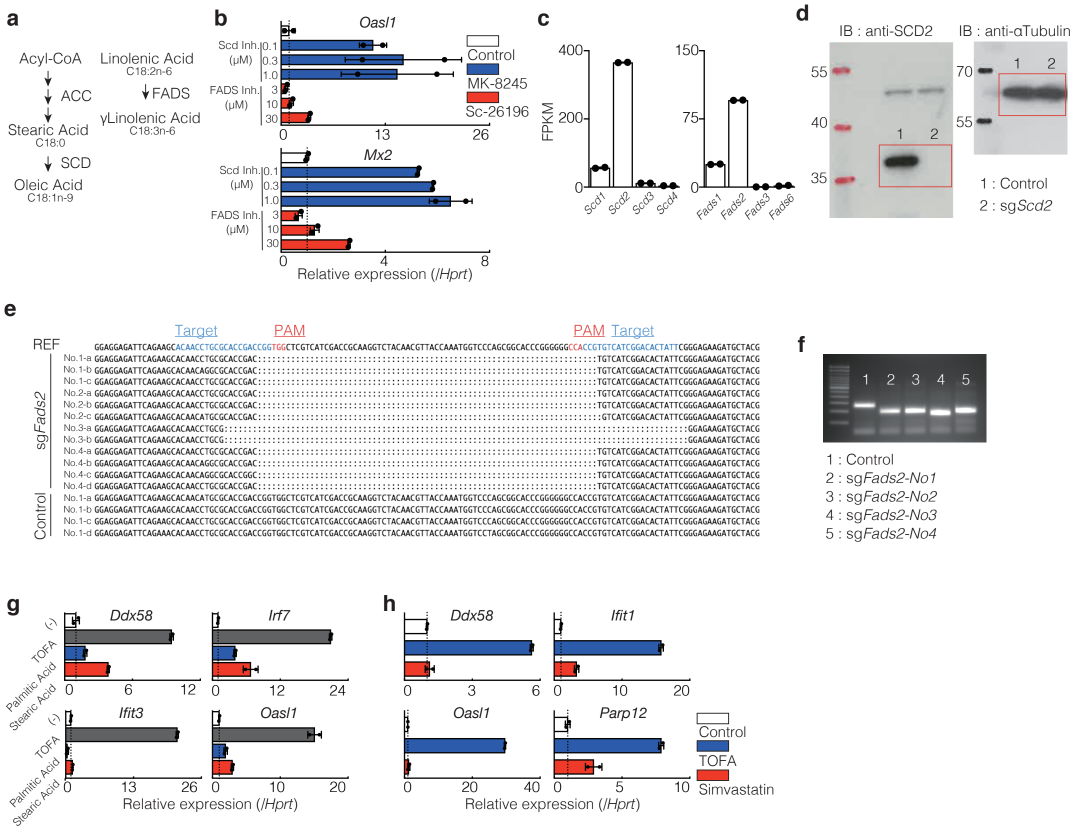

Supplementary Fig.4 Inhibition of MUFA synthesis is important for the induction of ISGs in CD4⁺ T cells related to Fig.4.

a, Scheme of fatty acid synthesis pathway is shown. **b**, qRT-PCR analyses of the relative expression of ISGs in Th1 cells treated with indicated concentration of SCD inhibitor (MK-8245) or FADS inhibitor (SC-26196) for 72 hours. Relative expression (normalized to *Hprt*) with SD is shown. **c**, Expression levels of *Scd* and *Fads* family in Th1 cells were shown as FPKM. Data used in this Figure is same as Fig. 1**a** (n=2 per each group biologically independent sample). **d**, Western blot analysis of SCD2 from control and sg*Scd2* Th1 cells. Two independent

experiments for each group were performed with similar results. (n=2 per each group biologically independent sample). **e**, Deletion patterns of the *Fads2* locus in control EL4 cells and sg*Fads2*-treated EL4 cells were shown. Reference (REF) sequence is shown on top of clonal sequences from each population with sgRNA target (blue) and PAM (red) sequences indicated. Black dashes denote deleted bases. **f,** Sequence of *Fads2* was amplified by PCR. **g**, qRT-PCR analyses of the relative expression of ISGs in Th1 cells treated with 10 µM TOFA, 30 µM palmitic acid or 10 µM stearic acid for 72 hours. Relative expression (normalized to *Hprt*) with SD is shown. **h**, qRT-PCR analyses of the relative expression of ISGs in Th1 cells treated with 10 µM TOFA, 1 µM simvastatin for 72 hours. Relative expression (normalized to *Hprt*) with SD is shown. More than two independent experiments were performed with similar results (**d = 2, b, g, h = 3**).

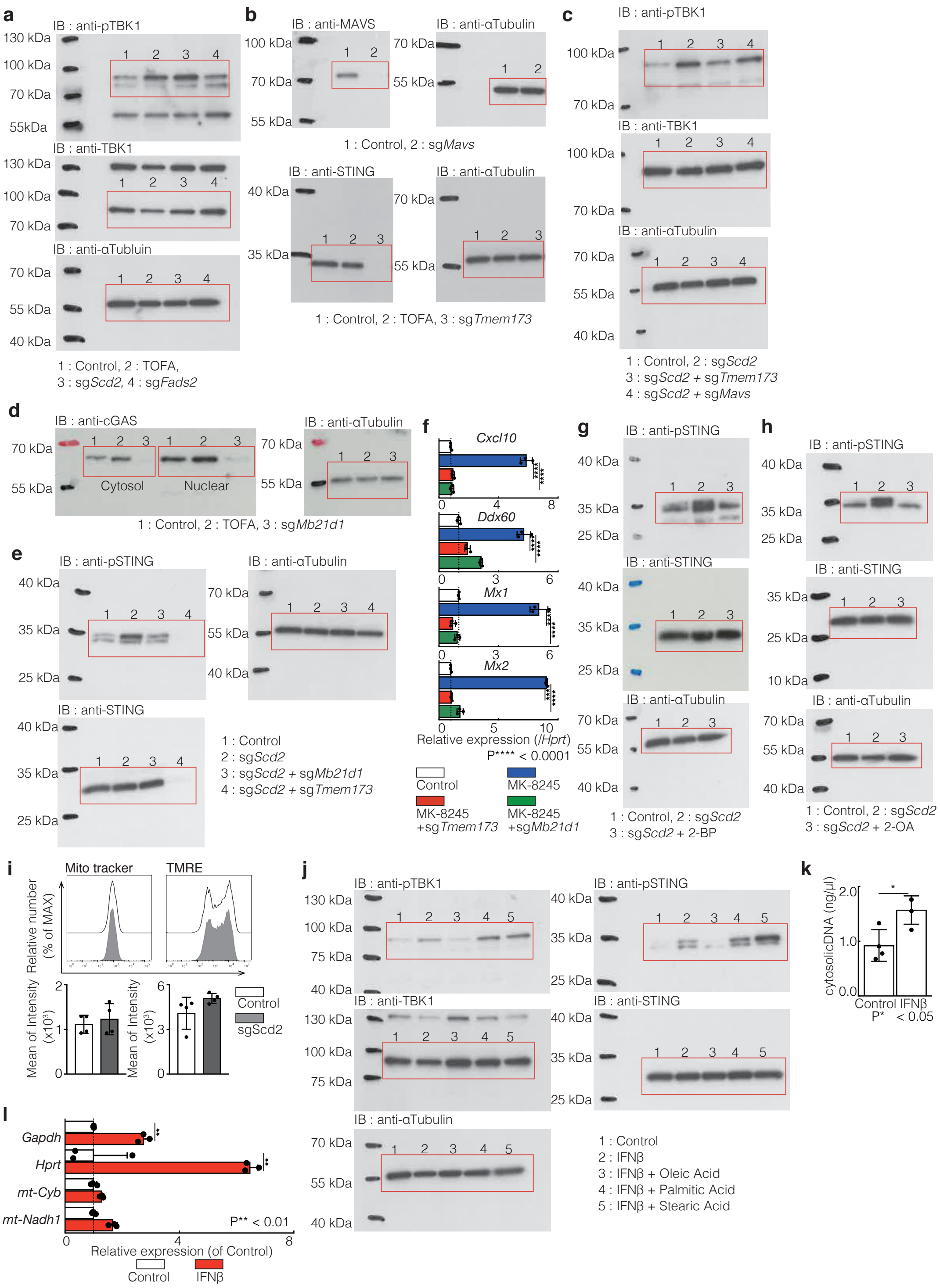

Supplementary Fig.5 CRISPR-Cas9 mediated genome edition occurs decreased protein expression related to Fig.5.

a, Uncropped scans of western blots were shown with labeled and molecular weight marker. **b-e,** Uncropped scans of western blots were shown with labeled and molecular weight marker. **f**, qRT-PCR analyses of the relative expression of ISGs in control Th1 cells, sg*Tmem173* cells or sg*Mb21d1* Th1 cells treated with or without MK-8245 for 72 hours. Relative expression (normalized to *Hprt*) with SD is shown. **g, h**, Uncropped scans of western blots were shown with labeled and molecular weight marker. **i**, Flow cytometry analyzing of mitochondrial mass (Mito tracker) and membrane potential (TMRE) in control or sg*Scd2* Th1 cells. **j**, Uncropped scans of western blots were shown with labeled and molecular weight marker. **k**, The amount of cytosolic DNA was measured by Qubit Fluorometer. Data are means ± SD. **l,** qRT-PCR analyses of the relative expression of genomic DNA and mitochondrial DNA in cytosolic DNA derived from control Th1 or IFNβ-treated Th1 cells. Relative expression (normalized to control Th1) with SD is shown. More than two independent experiments were performed with similar results (**f** = 2, **a-e, g-j,** and **l** = 3, **k** =4).

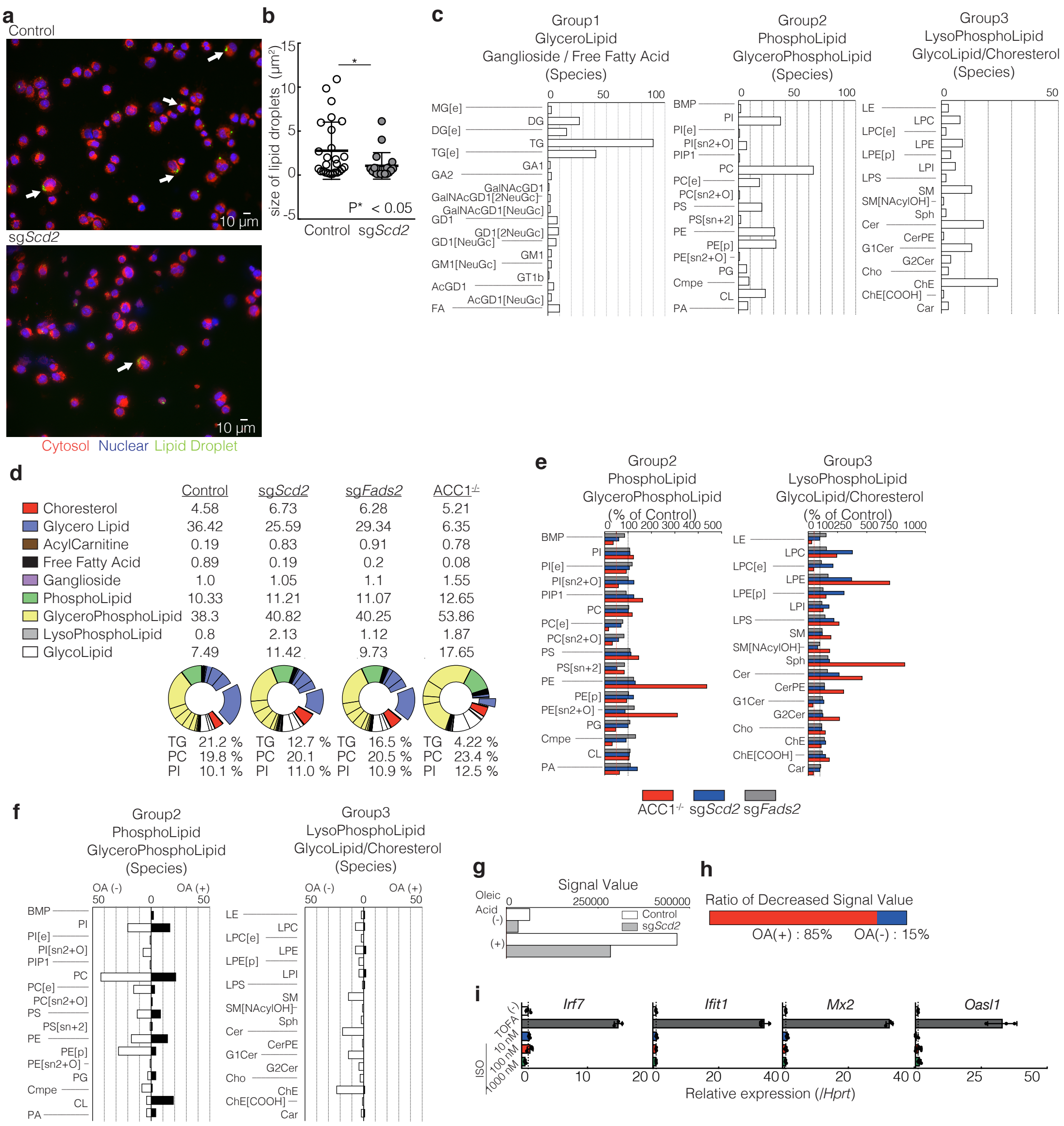

Supplementary Fig.6 Abrogation of fatty acid synthesis pathway changes in the lipid composition related to Fig.6.

a, Wide angle of image was shown related to Fig.6**a. b**, Graph shows the summary data of LD size related to supplementary Fig. 6a. **c,** Lipids detected in the lipidomic analysis were categorized into groups and the types of lipids measured are shown. **d,** Pie chart showed that the ratio of

lipid species in sg*Scd2*, sg*Fads2* and ACC1$^{-/-}$ Th1 cells compared to control Th1 cells. The species of molecular lipid in each lipid classes detected by lipidomics analysis were shown. **e,** Lipidomics analysis revealed the relative contents of molecular lipid species in ACC1$^{-/-}$, sg*Scd*2, and sg*Fads*2 Th1 cells compared to control Th1 cells. Normalized values are shown here. **f**, Graph shows the number of lipid types classified by the presence or absence of oleic acid. **g,** Graph shows the sum of TG signal values of control cells and sg*Scd2* Th1 cells with and without OA. **h,** Graph shows ratio of signal values of TG reduced in sg*Scd2* Th1 cells compared to control, with and without OA. **i,** qRT-PCR analyses of the relative expression of ISGs in Th1 cells treated with 10 μM TOFA or indicated concentration of ISO for 72 hours. Relative expression (normalized to *Hprt*) with SD is shown. More than two independent experiments were performed with similar results.

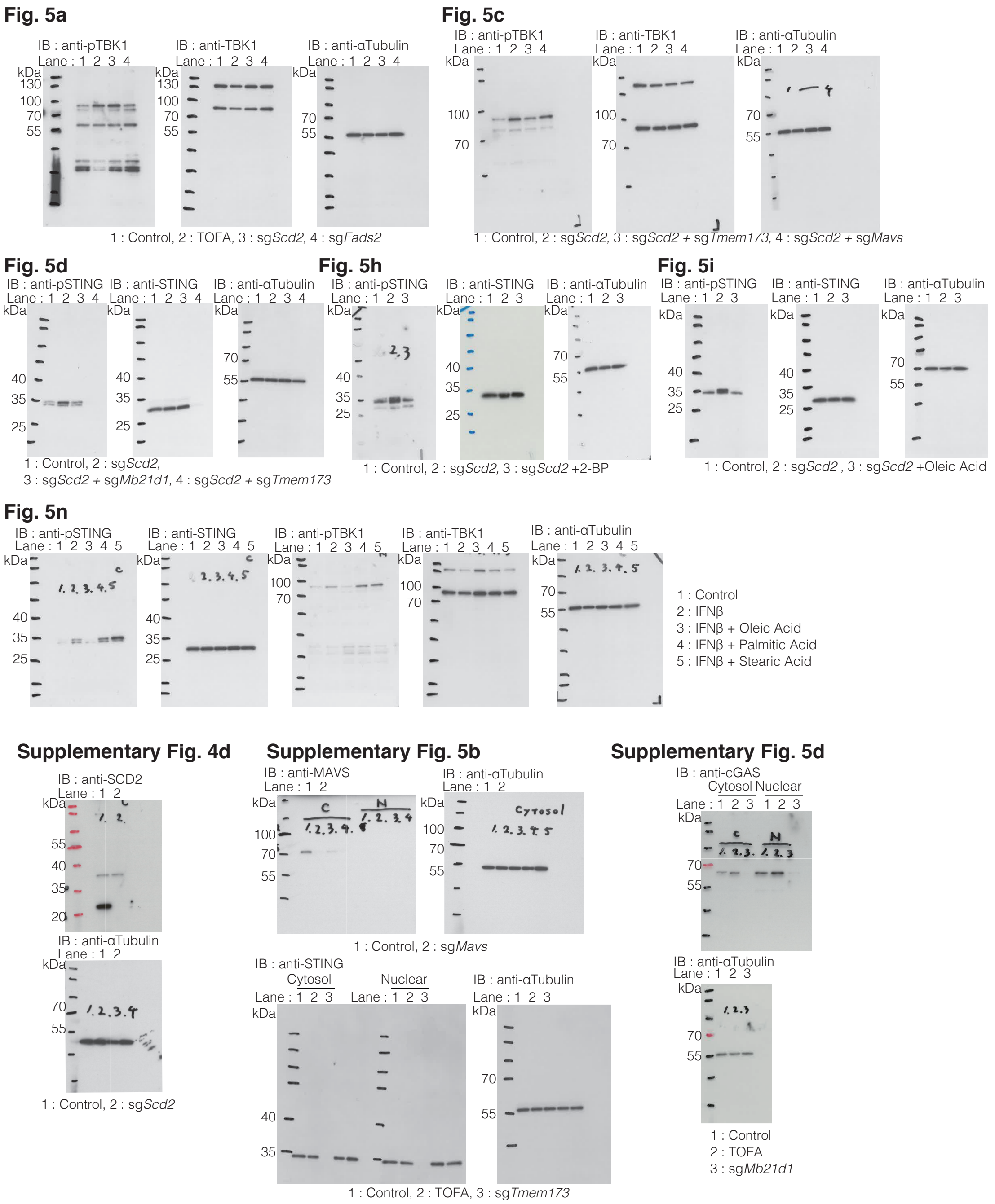

Supplementary Fig.7 Uncropped scans of western blots are shown with labeled and molecular weight ladder.
