# Supplementary material for: SCD2-mediated monounsaturated fatty acid metabolism regulates cGAS-STING-dependent type I IFN responses in CD4+ T cells
Source: Commun Biol. 2021 Jun 29;4:820. doi: 10.1038/s42003-021-02310-y (PMC8242023; doi:10.1038/s42003-021-02310-y)
Supplement: Supplementary file 3 — Description of Additional Supplementary Files [file 42003_2021_2310_MOESM3_ESM.pdf]

Description of Additional Supplementary Files

File name: Supplementary Data 1

Description: Source data for the graphs presented in Main and Supplementary figures.

File name: Supplementary Data 2

Description: Source data of lipidomics data including the description of each data set
