# Supplementary material for: SCD2-mediated monounsaturated fatty acid metabolism regulates cGAS-STING-dependent type I IFN responses in CD4+ T cells
Source: Commun Biol. 2021 Jun 29;4:820. doi: 10.1038/s42003-021-02310-y (PMC8242023; doi:10.1038/s42003-021-02310-y)
Supplement: Supplementary file 6 — Reporting Summary [file 42003_2021_2310_MOESM6_ESM.pdf]

nature research

Corresponding author(s): Yusuke Endo

Last updated by author(s): Apr 28, 2021

Reporting Summary

Nature Research wishes to improve the reproducibility of the work that we publish. This form provides structure for consistency and transparency in reporting. For further information on Nature Research policies, see our Editorial Policies and the Editorial Policy Checklist.

Statistics

For all statistical analyses, confirm that the following items are present in the figure legend, table legend, main text, or Methods section.

n/a	Confirmed	
☐	☒	The exact sample size (n) for each experimental group/condition, given as a discrete number and unit of measurement
☐	☒	A statement on whether measurements were taken from distinct samples or whether the same sample was measured repeatedly
☐	☒	The statistical test(s) used AND whether they are one- or two-sided<br>*Only common tests should be described solely by name; describe more complex techniques in the Methods section.*
☐	☒	A description of all covariates tested
☐	☒	A description of any assumptions or corrections, such as tests of normality and adjustment for multiple comparisons
☐	☒	A full description of the statistical parameters including central tendency (e.g. means) or other basic estimates (e.g. regression coefficient) AND variation (e.g. standard deviation) or associated estimates of uncertainty (e.g. confidence intervals)
☐	☒	For null hypothesis testing, the test statistic (e.g. F, t, r) with confidence intervals, effect sizes, degrees of freedom and P value noted<br>*Give P values as exact values whenever suitable.*
☒	☐	For Bayesian analysis, information on the choice of priors and Markov chain Monte Carlo settings
☒	☐	For hierarchical and complex designs, identification of the appropriate level for tests and full reporting of outcomes
☒	☐	Estimates of effect sizes (e.g. Cohen's d, Pearson's r), indicating how they were calculated

Our web collection on statistics for biologists contains articles on many of the points above.

Software and code

Policy information about availability of computer code

Data collection	FACSChorus v1.3, FACSDiva v8.0.1.1, StepOneSoftware v2.3.
Data analysis	FlowJo v10.4, CHOPCHOP 3.0.0, Prism v7, DAVID v6.8, GSEA v3.0, Bowtie v0.12.8, TopHat v1.3.2, Cufflinks v2.0.2., R v 3.6.0.

For manuscripts utilizing custom algorithms or software that are central to the research but not yet described in published literature, software must be made available to editors and reviewers. We strongly encourage code deposition in a community repository (e.g. GitHub). See the Nature Research guidelines for submitting code & software for further information.

Data

Policy information about availability of data

All manuscripts must include a data availability statement. This statement should provide the following information, where applicable:

- Accession codes, unique identifiers, or web links for publicly available datasets
- A list of figures that have associated raw data
- A description of any restrictions on data availability

All RNA-seq generated in this study will be deposited in Gene Expression Omnibus (GEO). The data that support the findings of this study are available from corresponding author upon reasonable request.

Field-specific reporting

Please select the one below that is the best fit for your research. If you are not sure, read the appropriate sections before making your selection.

☒ Life sciences ☐ Behavioural & social sciences ☐ Ecological, evolutionary & environmental sciences

For a reference copy of the document with all sections, see nature.com/documents/nr-reporting-summary-flat.pdf

Life sciences study design

All studies must disclose on these points even when the disclosure is negative.

Sample size	Sample size was chosen according to standard practices in the field.
Data exclusions	For lipidomics analysis, we excluded lipid molecules that showed peak below blank signal value.
Replication	We considered all of data were successfully reproduced by each attempt if similar results were obtained from at least 2 independent experiments.
Randomization	For in vivo experiments, mice of similar ages and sex were randomly used for all the experiments reported. In vitro experiments were all performed in parallel.
Blinding	The investigation were not blinded to the identities of the samples because treatment and data collection were performed by the same people. All samples were collected and analyzed at the same time under same conditions. Blinding was not relevant for most in-vitro procedures as the readouts were automated and quantitative.

Reporting for specific materials, systems and methods

We require information from authors about some types of materials, experimental systems and methods used in many studies. Here, indicate whether each material, system or method listed is relevant to your study. If you are not sure if a list item applies to your research, read the appropriate section before selecting a response.

Materials & experimental systems

n/a	Involved in the study	
☐	☒	Antibodies
☐	☒	Eukaryotic cell lines
☒	☐	Palaeontology and archaeology
☐	☒	Animals and other organisms
☐	☒	Human research participants
☒	☐	Clinical data
☒	☐	Dual use research of concern

Methods

n/a	Involved in the study	
☒	☐	ChIP-seq
☐	☒	Flow cytometry
☒	☐	MRI-based neuroimaging

Antibodies

Antibodies used	The reagents used in this study were as follows: the FITC- and BV650-conjugated anti-CD4 (FITC; 116004, RM4-4, 1μg ml-1 and BV650; 563747, RM4-5, 1μg ml-1); FITC-conjugated anti-CD19 (115506, 6D-5, 1μg ml-1); Alexa 647- and BV421-conjugated anti-CD317 (Alexa 647; 127014, 927, and BV421; 127203, 927, 1ug ml-1); PE-conjugated anti-TCRβ (109208, H57-597, 1μg ml-1); PE/Cy7-conjugated anti-B220 (103222, RA3-6B2, 1μg ml-1); agonistic anti-CD3 (100340, 145-2C11, 1μg ml-1), agonistic anti-CD28 (102102, 37.51, 1μg ml-1), anti-IL-4 (504122, 11B11, 1μg ml-1), anti-IFNγ (505707, R4-6A2, 1μg ml-1) were purchased from BioLegend (San Diego, CA).<br>APC-conjugated anti-CD8a (553035, 53-6.7, 1μg ml-1); BV786-conjugated anti-CD45 (564225, 30-F11, 1μg ml-1); anti-IL4 (554386, BVD4-1D11, 1μg ml-1) were purchased from BD biosciences. FITC-conjugated anti-CD45.1 (11-0453-82, A20, 1μg ml-1); APC-conjugated anti-CD45.2 (17-0454-82, 104, 1μg ml-1) were purchased from eBiosciences.<br>PE-conjugated anti-IRF7 (12-5829-82, MNGPKL, 1μg ml-1); alpha-Tublin (62204, DM1A, 1ng ml-1) were purchased from Thermo Fisher Scientific.<br>anti-cGAS (31659, D3080, 1:2,000); anti-MAVS (4983, Rodent specific, 1:2,000); anti-STING(13647, D2P2F, 1:2,000); anti-phospho STING(Ser365)(62912, D1C4T, 1:2,000); anti-TBK1/NAK (3504, D1B4, 1:2,000); anti-phosphoTBK1/NAK(Ser172) (5483, D52C2, 1:2,000);HRP-conjugated anti-Rabbit IgG (7074, polyclonal, 1:5,000) were purchased from Cell Signaling.<br>anti-Scd2 (sc-518034, H-12, 1:2,000) were purchased from Santa cruz.<br>HRP-conjugated anti-Mouse IgG (NA931, polyclonal, 1:2,000) were purchased from GE Healthcare.
Validation	Antibodies were chosen based on the validation statements for species (mouse) and applications (FACS or WB) on the manufacturer's website. For example, according to the manufacture's website (https://www.biolegend.com/nl-be/products/fitc-anti-mouse-cd4-antibody-475), the anti-CD4-FITC antibody was validated at least for the use of FACS analysis of mouse cells.

Eukaryotic cell lines

Policy information about cell lines

Cell line source(s)	MLE-15 (Mouse Lung Epithelial Cell)
Authentication	MLE-15 were purchased from Applied Biological Material Inc.
Mycoplasma contamination	Mycoplasma contamination was not found in MLE-15 cells.
Commonly misidentified lines (See ICLAC register)	*Name any commonly misidentified cell lines used in the study and provide a rationale for their use.*

Animals and other organisms

Policy information about studies involving animals; ARRIVE guidelines recommended for reporting animal research

Laboratory animals	The animals used in this study were backcrossed to C57BL/6 mice 10 times. Acacafl/fl mice were crossed with CD4-cre mice (Jackson Laboratory) and were maintained on a C57BL/6 background. Ly5.1 mice were purchase from Sankyo Laboratory. C57BL/6 mice were purchased from Clea Inc., Tokyo, Japan. The sex-matched mice were used at 6-8 weeks of ages and maintained under specific-pathogen-free (SPF) conditions.
Wild animals	The study did not involve wild animals.
Field-collected samples	The study did not involve samples collected from the field.
Ethics oversight	The research proposals were reviewed by the ethics committee for animals as Chiba University (Registration number: #28-181), KAZUSA DNA Research Institute (Registration number: 30-1-002), and the Institutional Animal Care and Use Committee of the the National Institute of Infectious Disease Japan (Registration number: 118105-II).

Note that full information on the approval of the study protocol must also be provided in the manuscript.

Human research participants

Policy information about studies involving human research participants

Population characteristics	2 randomly selected healthy male and female donors.
Recruitment	Blood from healthy donors was obtained from KAZUSA DNA Research Institute and used in compliance with KAZUSA DNA Research Institute Administrative Panel for human subjects (authorization #2020-01). There was no selection bias.
Ethics oversight	KAZUSA DNA Research Institute

Note that full information on the approval of the study protocol must also be provided in the manuscript.

Flow Cytometry

Plots

Confirm that:

☒ The axis labels state the marker and fluorochrome used (e.g. CD4-FITC).

☒ The axis scales are clearly visible. Include numbers along axes only for bottom left plot of group (a 'group' is an analysis of identical markers).

☒ All plots are contour plots with outliers or pseudocolor plots.

☒ A numerical value for number of cells or percentage (with statistics) is provided.

Methodology

Sample preparation	See Methods
Instrument	Becton Dickson 3-laser FacsCellesta for analysis and Melody for cell sorting
Software	BD FACSDiva software for data acqisition and analyzed using Flowjo software v10.4. data was graphed using Prism 7 (Graphpad)
Cell population abundance	The purities of sorted cell populations were consistently > 90-98%.

Gating strategy

The gating strategy is shown in each Figure.

☒ Tick this box to confirm that a figure exemplifying the gating strategy is provided in the Supplementary Information.
