# Supplementary material for: SCD2-mediated monounsaturated fatty acid metabolism regulates cGAS-STING-dependent type I IFN responses in CD4+ T cells
Source: Commun Biol. 2021 Jun 29;4:820. doi: 10.1038/s42003-021-02310-y (PMC8242023; doi:10.1038/s42003-021-02310-y)
Supplement: Supplementary file 1 — Peer Review File [file 42003_2021_2310_MOESM1_ESM.pdf]

Editorial Note: This manuscript has been previously reviewed at another journal that is not operating a transparent peer review scheme. This document only contains reviewer comments and rebuttal letters for versions considered at Communications Biology.

REVIEWERS' COMMENTS:

Reviewer #3 (Remarks to the Author):

This study represents a novel and important contribution to the field of immunometabolism which deserves to be published.
